# Stakeholder-informed pre-implementation refinement of a pharmacy-led pre-exposure prophylaxis (PrEP) service delivery model in Malaysia

**DOI:** 10.1371/journal.pone.0357920

**Published:** 2026-09-08

**Authors:** Yan Nee Gan, Sin How Lim, Frederick L. Altice, Sharifah Khadijah Syed Razif, Mariani Ahmad Nizaruddin, Reena Rajasuriar, Sazali Basri, Joselyn Pang, Andrew Yap, Iskandar Azwa, Rafdzah Zaki

**Affiliations:** 1 Department of Social and Preventive Medicine, Faculty of Medicine, Universiti Malaya, Kuala Lumpur, Malaysia; 2 Malaysian Health Technology Assessment Section (MaHTAS), Ministry of Health, Putrajaya, Malaysia; 3 Centre of Excellence for Research in Infectious Diseases and AIDS (CERiA), Universiti Malaya, Kuala Lumpur, Malaysia; 4 Section of Infectious Disease, Department of Internal Medicine, Yale School of Medicine, New Haven, Connecticut, United States of America; 5 Division of Epidemiology of Microbial Diseases, Yale School of Public Health, New Haven, Connecticut, United States of America; 6 Center for Interdisciplinary Research on AIDS (CIRA), Yale University, New Haven, Connecticut, United States of America; 7 Department of Community and Pharmacy Practice, Faculty of Pharmacy, University of Cyberjaya, Cyberjaya, Selangor, Malaysia; 8 Department of Medicine, Faculty of Medicine, Universiti Malaya, Kuala Lumpur, Malaysia; 9 Infectious Diseases Unit, Department of Medicine, Faculty of Medicine, Universiti Malaya, Kuala Lumpur, Malaysia; 10 The Red Clinic, Petaling Jaya, Selangor, Malaysia; 11 Centre for Epidemiology and Evidence-Based Practice (CEBP), Universiti Malaya, Kuala Lumpur, Malaysia; Universiti Monash Malaysia: Monash University Malaysia, MALAYSIA

## Abstract

**Introduction:**

In 2024, only 3,451 individuals accessed free oral HIV pre-exposure prophylaxis (PrEP) at government clinics in Malaysia, despite an estimated 354,000 key population members who could benefit from PrEP. To inform pre-implementation refinement of a pharmacy-led PrEP service delivery model, we explored stakeholder perspectives on implementing the model within private community pharmacies in Malaysia.

**Materials and methods:**

A one-day stakeholder consultation was conducted in April 2023 in Kuala Lumpur with 28 stakeholders representing community pharmacies, professional societies, telemedicine providers, non-governmental organizations (NGOs), and HIV implementation science researchers. The meeting began with a review of preliminary findings from formative qualitative research involving key populations, community pharmacists, and PrEP prescribers. Through facilitated discussions, stakeholders identified short- and long-term solutions to implementation barriers. Stakeholder inputs from discussions and structured worksheets were synthesized descriptively to identify recurring implementation considerations that informed refinement of the pilot model.

**Results:**

Key implementation considerations included enhancing awareness and demand generation, ensuring privacy, confidentiality, and affordability, integrating HIV self-testing, using digital checklist-based assessments, simplifying laboratory testing requirements, developing practical workflows supported by a reference guide, strengthening pharmacist training, and fostering cross-sector collaboration. Identified training needs included PrEP fundamentals, eligibility assessment for initiation and continuation, referral procedures, and scenario-based learning. Key competencies included knowledge of HIV and sexually transmitted infections, risk assessment, professional conduct, and person-centered communication. Six study sites were selected, with preparation requirements including HIV self-test kits, PrEP medications, informational materials, trained pharmacists, telemedicine subscriptions, and nearby referral clinics. The consultation informed refinements to the implementation plan.

**Conclusions:**

This stakeholder consultation informed refinement of a pharmacy-led PrEP service delivery model for pilot implementation in Malaysia and identified practical implementation considerations related to privacy, affordability, HIV self-testing, physician-pharmacist coordination, training, and referral systems. The findings may inform future implementation planning for similar urban, private-sector PrEP delivery models.

## Introduction

In 2015, Malaysia incorporated oral HIV pre-exposure prophylaxis (PrEP) into its National Strategic Plan to End AIDS by 2030, supported by the Malaysian Consensus Guidelines on Antiretroviral Therapy published in 2017 [1,2]. Tenofovir-based oral PrEP is available free of charge at 31 government clinics and on an out-of-pocket basis at 15 private facilities, yet uptake remains low [3,4]. In 2024, Malaysia reported 3,185 new HIV diagnoses [5]. HIV prevalence remains disproportionately high among the estimated 354,000 key population members in Malaysia, including 12.9% among men who have sex with men (MSM), 7.5% among people who inject drugs (PWID), and 5.9% among transgender women [6,7].

Although free PrEP services have been available through government clinics since 2023, only 3,451 individuals accessed these services in 2024, representing a small fraction of the estimated population who could benefit from PrEP [5]. This persistent gap suggests that structural, social, and service delivery barriers continue to constrain PrEP access in Malaysia.

The World Health Organization recommends differentiated service delivery (DSD) models for HIV prevention, including pharmacy- and telehealth-based PrEP delivery to reduce stigma, improve convenience, and expand access for populations underserved by traditional clinic-based services [8]. This shift aligns with the demedicalization of PrEP, whereby services are delivered outside traditional healthcare settings to improve accessibility [9]. The pharmacy-led model, successful in several countries, offers accessible, personalized care and presents a less stigmatized access point for at-risk populations [10–16]. In Malaysia, some MSM reported a preference for accessing PrEP through pharmacies [17], likely reflecting the frequent and familiar use of community pharmacies among pharmacy users, who reported an average of 31 visits annually [18]. With extended operating hours and wide geographic distribution, Malaysian community pharmacies are well-positioned to serve as additional PrEP access points, however, several barriers remain before wide-scale adoption of this DSD model [15,16,19].

Implementing pharmacy-led PrEP services requires alignment with local policies, infrastructure, workforce capacity, and referral systems [20]. Financial sustainability is also critical in private sector settings, where service delivery entails additional time and opportunity costs [15]. Multi-level stakeholder engagement is essential to address system and provider barriers, align expectations, and support implementation [21].

Stakeholder engagement is a core implementation strategy for adapting evidence-based practices to real-world contexts. As demonstrated in Kenya and Nigeria, collaborative consultations help align service models with local workflows, regulatory structures, and client needs [14,22]. By bringing together implementers, providers, and affected communities, these processes help align service models with local contexts, inform implementation planning, and strengthen stakeholder ownership, all of which may support future implementation and scale-up of pharmacy-led PrEP services in Malaysia.

This consultation engaged multi-sector stakeholders to inform refinement of a pharmacy-led PrEP service delivery model in Malaysia. While pharmacy-led PrEP services have been explored in other settings, little is known about how such services can be adapted and implemented within Malaysia’s legal, regulatory, and sociocultural context. This stakeholder consultation builds on prior formative research and provides context-specific insights to inform implementation and service refinement.

## Materials and methods

### Implementation context

The Klang Valley, an urban region encompassing Selangor, Kuala Lumpur, and Putrajaya, accounts for approximately 46% of new HIV infections reported nationwide in 2024 [5]. This region has a dense healthcare infrastructure with approximately 16 public hospitals, 114 public clinics, and more than 3,000 private general practitioner (GP) clinics [19]. Of the 3,084 community pharmacies nationwide, around one-third are located in the Klang Valley, highlighting their accessibility within the region [19].

Although Malaysia’s public healthcare facilities provide subsidized medical care, community pharmacies are widely used for medication supply, health supplements, and advice on minor health concerns [23]. Many pharmacies also offer health screening services (e.g., blood pressure, blood glucose, and cholesterol testing) and offer professional consultations on chronic conditions such as hypertension and diabetes, positioning them as accessible care points [23].

Pharmacists are authorized to dispense prescription medications and provide counseling under the Registration of Pharmacists Act 1951 and the Poisons Act 1952, regulated by the Pharmacy Board of Malaysia under Ministry of Health Malaysia (MOH) [24]. Oral tenofovir disoproxil fumarate/emtricitabine (TDF/FTC), recommended for HIV PrEP in the Malaysian Consensus Guidelines on ART [25], is classified as a Group B Poison and requires a prescription by a registered medical practitioner prior to dispensing [24]. Recent expansion of telehealth services has enabled electronic prescribing and closer collaboration between pharmacists and physicians, facilitating streamlined access to medications [26]. This evolving ecosystem supports task-sharing approaches for PrEP delivery while remaining aligned with regulatory requirements.

### Stakeholder consultation meeting

This stakeholder consultation was conducted as a minimal-risk component of an Institutional Review Board (IRB)-approved implementation research study designed to inform the development and refinement of the implementation strategy for a pharmacy-led PrEP service delivery model. As part of the Exploration–Preparation–Implementation–Sustainment (EPIS) Preparation phase, we convened stakeholders relevant to both the inner context of participating pharmacies and the broader prescribing, telemedicine, professional, and community systems required for pharmacy-led PrEP delivery in a structured, one-day stakeholder consultation held on 15 April 2023 [27].The consultation was conducted in a hybrid format to enable broad attendance. During the session, stakeholder input was systematically collected through structured worksheets, facilitated group discussions, and field notes to identify context-specific implementation strategies and refine the pharmacy-led PrEP service delivery model for pilot implementation.

The consultation built on findings from the preceding exploratory phase, which involved in-depth interviews with key populations, community pharmacists, and PrEP prescribers. These findings identified barriers and facilitators to pharmacy-led PrEP delivery and informed the stakeholder discussions. Preliminary findings were presented to ground subsequent discussions and stakeholder consultation activities, informing refinement of service workflows, training requirements, and site preparation.

### Stakeholder selection

All invited stakeholders were informed of the objectives of the one-day stakeholder meeting, and participation was voluntary. Invitations were directed towards individuals with direct roles in PrEP delivery, clinical care, community engagement, or implementation who were interested in informing the development of the new pharmacy-led PrEP delivery model. Representation from diverse stakeholder groups enabled triangulation across operational, clinical, and community perspectives and mirrored consultation structures used in Kenya and Nigeria [14,22].

Twenty-eight stakeholders attended the meeting; 12 invited stakeholders declined due to scheduling conflicts. Stakeholders represented the community pharmacy implementation partner, telemedicine partner, PrEP prescribers, professional societies, implementation science researchers, and non-governmental organizations (NGOs) involved in HIV prevention and support services (Table 1). These NGOs serve key populations affected by HIV, including MSM, PWID, transgender women, and sex workers. Several NGO representatives were community health workers from these communities and contributed lived and/or professional perspectives during the consultation.

**Table 1:** Stakeholder groups and roles in the consultation.

Stakeholder group	Number	Role in the consultation
Community pharmacists (Alpro Pharmacy)	7	Provided perspectives on pharmacy operations, workflow, training and site readiness
Telemedicine representatives (DOC2US^®^)	4	Advised on teleconsultation, electronic prescribing and integration with the pharmacy workflow
PrEP prescribers (including two members of the Malaysian Society for HIV Medicine [MASHM])	6	Advised on clinical assessment, prescribing, monitoring and referral procedures
Malaysian Pharmacists Society (MPS) representatives	2	Provided professional and pharmacy-practice perspectives
HIV implementation science researchers	2	Provided implementation science and service-design perspectives
NGO representatives ^a^	7	Provided community engagement, HIV-prevention and key population service perspectives
**Total**	**28**	

Stakeholders were classified according to their primary stakeholder role. One telemedicine representative was also a PrEP prescriber but was classified under the telemedicine category. Two of the six PrEP prescribers were members of the Malaysian Society for HIV Medicine (MASHM).

^a^NGOs included the Malaysian AIDS Council, PT Foundation, Kuala Lumpur AIDS Support Services Society, SEED Foundation, and Persatuan Insaf Murni Malaysia.

Alpro Pharmacy, a large independent community pharmacy chain in Malaysia, was selected as the community pharmacy implementation partner based on its willingness to participate, operational capacity, and existing collaboration with DOC2US^®^. DOC2US^®^ was selected as the telemedicine partner based on its established digital health infrastructure, physician network, and willingness to support PrEP service delivery. NGOs were selected based on their involvement in HIV prevention services and willingness to collaborate. Representatives from the MOH Disease Control Division and Pharmacy Program were not invited in their official programmatic or regulatory capacities, as the consultation focused on implementation planning for a pilot pharmacy-led PrEP service model within private community pharmacies operating under existing regulatory frameworks. However, two MOH specialists participated as public-sector PrEP prescribers and were included within the prescriber stakeholder group rather than as MOH representatives.

### Meeting activities

The stakeholder consultation followed a structured sequence of activities and was facilitated by the lead researcher, who has experience in HIV prevention, implementation science, and community pharmacy research. The meeting commenced with a presentation of preliminary findings from the exploration phase, which involved semi-structured interviews with 18 individuals from key populations, 10 community pharmacists, and 10 PrEP prescribers, highlighting perceived barriers and facilitators of pharmacy-led PrEP services (S1 Appendix).

Stakeholders then participated in discussions to identify short- and long-term solutions to implementation barriers, defined as actionable strategies for the pilot study and longer-term system-level changes. Following the identification of proposed short- and long-term solutions, stakeholders and the research team discussed which short-term solutions should be incorporated into the pilot implementation, while solutions requiring broader policy, regulatory, or resource changes were retained as longer-term considerations. Inputs were documented using structured Google Docs worksheets (S2 Appendix) and organized according to the core components of the pharmacy-led PrEP service delivery model adapted from a Kenyan study [28]. These components included promotional activities and materials, HIV testing, assessment and counseling, prescribing by physician, dispensing, follow-up, oversight and referrals, and other operational considerations.

Subsequently, stakeholders reviewed the proposed pilot study procedures, care pathway, workflow, schedule of activities (S3 Appendix), and assessment tools (S4 Appendix). Training needs and competencies for community pharmacists were identified through a synchronous Mentimeter^®^ activity (Menti.com) [29]. Stakeholders were asked to identify the knowledge and competencies required for pharmacists to deliver PrEP services. The stakeholder recommendations informed the development of a pharmacist PrEP training program to support pilot implementation, which has been described and evaluated separately [30]. The meeting also included discussion of study site selection criteria and preparation requirements, leading to the finalization of six study sites. Promotional and educational materials for participant recruitment and service awareness were also reviewed and refined during the consultation but are not included as Supporting Information. The consultation concluded with a facilitator-led summary of the proposed implementation plan.

### Stakeholder input documentation

Stakeholder input was documented through worksheets, facilitated discussions, and field notes. Inputs on proposed solutions were captured in real time using structured Google Docs worksheets, complemented by open-text responses from the Mentimeter^®^ activity. Two members of the research team recorded field notes to document key discussion points, areas of agreement, and contextual observations. Discussions were also audio-recorded to support accurate documentation and verification of meeting notes. Verbatim transcripts were not produced. Where differing views arose, these were discussed during the consultation and reflected in the final recommendations. The facilitator summarized the recommendations discussed and confirmed them with stakeholders before proceeding to subsequent activities.

### Synthesis of stakeholder input

The consultation was designed to inform program implementation, and stakeholder inputs were synthesized to derive transferable insights relevant to pharmacy-led PrEP delivery. Inputs from discussion notes, completed worksheets, and Mentimeter^®^ responses were collated, reviewed, and synthesized descriptively by two members of the research team (YG and SS). Any differences in interpretation were discussed and resolved through consensus before finalization of the synthesized findings. The documented stakeholder inputs were not subjected to formal qualitative coding or thematic analysis, consistent with the formative purpose of the consultation. Stakeholder inputs were iteratively organized according to the core components of the pharmacy-led PrEP service delivery model, with a focus on proposed short- and long-term solutions. Findings informed refinement of the care pathway, pharmacist training requirements, site preparation needs, and overall implementation plan.

Descriptive synthesis enabled identification of recurring implementation considerations and areas for service refinement. Areas of convergence were identified when similar issues, recommendations, or implementation needs were raised across multiple consultation activities, including discussions on proposed solutions, pilot procedures, workflow design, assessment tools, training requirements, and site readiness. Synthesized findings were subsequently reviewed by YG and SS, together with IA, a senior clinician-researcher with expertise in HIV prevention who participated in the stakeholder consultation, to ensure consistency with the documented stakeholder inputs. A summary report was circulated to all 28 stakeholders for feedback and accuracy checking. One stakeholder acknowledged receipt and did not request changes; no other stakeholders responded. Because of the limited response, this circulation was not interpreted as formal stakeholder validation.

### Ethical approval

All aspects of this implementation research program, including the stakeholder consultation described here, were reviewed and approved by the University of Malaya Medical Centre Medical Research Ethics Committee (MREC ID: 20221026−11645) in alignment with the Helsinki Declaration. The consultation was conducted as an implementation-informed stakeholder engagement activity to support pre-implementation planning, rather than as a formal qualitative study intended to generate theory-driven findings. Participants were informed of the purpose of the consultation, including its role in informing the development and implementation of a pharmacy-led PrEP service model as part of a broader research program. The consultation involved professional stakeholders contributing in their organizational or professional roles and did not involve collection of personal or sensitive individual-level data. Participation was voluntary, and participants agreed to be acknowledged for their contributions.

Stakeholder input was documented through structured worksheets, facilitated discussions, and field notes. No personally identifiable information was recorded, and all inputs were anonymized at the point of documentation. Findings are reported in aggregate form to preserve confidentiality. Participants were acting in their professional capacities as subject-matter experts and stakeholders in PrEP service delivery. Participants were reimbursed for travel-related expenses and provided with refreshments; no financial incentives were provided, and participation or non-participation had no impact on professional relationships.

## Results

Stakeholders proposed short- and long-term solutions to address barriers to the pharmacy-led PrEP service (Table 2). Short-term solutions were proposed as potentially suitable for incorporation during the pilot, whereas long-term solutions involved broader system-level changes. In addition to the proposed solutions, synthesis of stakeholder inputs from discussions on service delivery, workflow design, pilot procedures, assessment tools, training requirements, and site preparation identified recurring implementation considerations that informed refinement of the pilot study procedures, care pathway, workflow, assessment tools, training program, and implementation plan. Because no formal prioritization or ranking exercise was conducted, these considerations should be interpreted as recurring stakeholder-identified implementation needs rather than as a ranked set of priorities.

**Table 2:** Stakeholder-proposed short-term and long-term solutions to address perceived barriers identified by key populations, community pharmacists, and PrEP prescribers, organized according to the core components of the pharmacy-led PrEP service delivery model.

Core components of the pharmacy-led PrEP service delivery model	Perceived barriers identified during the exploratory phase among key populations, community pharmacists	Solutions proposed by stakeholders	
		Short-term	Long-term
**Promotional activities & materials**	• Poor awareness about PrEP • Poor awareness about the pharmacy-led PrEP service • Lack of demand • Unfamiliarity / uncertainty regarding a new service	• √ Promote the service and disseminate PrEP information through websites, social media, telemedicine platforms, and clinics. • √ List pharmacies on the My PrEP Locator – a web-based directory for PrEP providers in Malaysia. • √ Implement digital appointment booking. • √ Display PrEP service stickers in pharmacies. • √ Partner with NGOs to support demand generation and client referrals. • √ Collaborate with condom suppliers to provide free condoms. • √ Provide referral incentives for CHWs and client peers. • Consider placing CHWs in pharmacies to support testing and enrolment.	• Develop public-private partnerships with the MOH for reimbursement. • Establish a community-endorsed PrEP pharmacy rating system.
**HIV testing**	• Concerns about privacy and confidentiality • High costs / affordability • Accuracy of oral fluid HIV self-test kits • Concerns about the ability to support clients with reactive test results	• √ Provide a private consultation room. • √ Integrate HIV self-testing into the service. • √ Use HIV self-test kits approved by the Medical Device Authority. • √ Educate clients on HIV testing, test accuracy and pharmacy services. • √ Partner with nearby clinics and laboratories for confirmatory testing. • √ Provide pre-test information and post-test counseling. • √ Include HIV testing, counseling, and linkage guidance in pharmacist training and reference guides. • √ Partner with CHWs for psychosocial support. • √ Establish a pharmacist support helpline. • √ Partner with CHWs to conduct community-based testing at pharmacies. • √ Conduct mid-term evaluations to identify implementation gaps. • Offer online consultations during peak hours.	• Develop HIV self-testing guidelines for pharmacists. • Maintain ongoing training and mentorship programs. • Establish accredited training for community pharmacists in community-based testing and voluntary counseling and testing. • Partner with NGOs or MOH to offer free / subsidized HIV testing.
**Assessment and counseling**	• Concerns about privacy and confidentiality • Concerns about pharmacists’ professionalism and capabilities • Concerns about the ability to provide effective counseling • Lack of knowledge / skills / experience • Insufficient training • Time constraints • Profitability / opportunity cost • Increased workload • Lack of manpower • Uncertainty about client disclosure / clients withholding information • Lack of laboratory services • Concerns about kidney function monitoring • Concerns about potential non-adherence to guidelines and pilot study procedures • Concerns about documentation of client record	• √ Provide a private counseling space. • √ Restrict data access to assigned pharmacists and physicians. • √ Implement services in pharmacies with ≥ 2 full-time pharmacists. • √ Schedule appointments on specific days / times. • √ Provide comprehensive in-person PrEP training. • √ Provide a reference guide for assessments, counseling topics, and required tests. • √ Ensure assessments align with national guidelines. • √ Simplify laboratory testing requirements. • √ Inform clients of mandatory HIV screening and baseline renal function testing. • √ Partner with clinics and laboratories for required testing. • √ Offer routine health screening at pharmacies. • √ Use digital assessment tools for documentation. • √ Establish pharmacist support channels. • √ Partner with infectious disease physicians for guidance and referrals. • Offer online risk assessment before pharmacy visits.	• Develop an online PrEP course for pharmacists. • Establish accredited PrEP training for pharmacists. • Provide a standardized toolkit or reference guide to support scale-up of pharmacy-led PrEP implementation. • Implement continuous monitoring and quality assurance. • Integrate HIV prevention and counseling into undergraduate curricula.
**Prescribing by physician**	• Concerns about potential non-adherence to guidelines and pilot study procedures • Concerns about documentation of client record	• √ Ensure proper documentation in the electronic record system. • √ Ensure client and provider access to assessment information.	• Explore pharmacist prescribing to support task-sharing and demedicalization, including the potential down-regulation of oral TDF/FTC under the Poisons Act.
**Dispensing**	• High costs / affordability • Profitability / opportunity cost • Concerns about potential non-adherence to guidelines and pilot study procedures	• √ Dispense PrEP only with a valid prescription, in accordance with the Poisons Act. • √ Provide medication rebates during the pilot.	• Negotiate lower PrEP prices with suppliers. • Engage insurers to cover PrEP. • Advocate for MOH reimbursement and tax relief for PrEP and HIV test kits.
**Follow-up**	• Concerns about pharmacists’ professionalism and capabilities • Concerns about the ability to support clients with reactive test results • Concerns about the ability to provide effective counseling • Concerns about PrEP use (side effects, kidney impairment) • Concerns about documentation of client record • Concerns about potential loss to follow up	• √ Provide comprehensive PrEP counseling during initiation and follow-up. • √ Advise clients to have annual physician follow-up. • √ Encourage clients to provide laboratory results during follow-up. • √ Ensure proper documentation in electronic systems. • Offer tele-pharmacy consultation and delivery options.	• Implement a unique ID system that allows clients to access PrEP services across different pharmacies and supports continuity of care.
**Oversight &** **Referrals**	• Concerns about pharmacists’ professionalism and capabilities • Concerns about the ability to support clients with reactive test results • Concerns about the ability to provide effective counseling • Concerns about documentation of client record • Concerns about potential non-adherence to guidelines and pilot study procedures	• √ Establish a referral network with physicians and CHWs. • √ Provide comprehensive PrEP training. • √ Provide a reference guide covering assessments, counseling, required tests, and referral guidance.	• Implement continuous monitoring and quality systems.
**Others**	• Stigma and discrimination • Personal / religious beliefs • Unwillingness of pharmacists to provide service • Discomfort among staff and customers	• √ Provide a private counseling room. • √ Engage pharmacists willing to provide the service. • √ Increase staff awareness of PrEP and ethical responsibilities. • √ Include SOGIE content in training. • √ Conduct sensitization workshops for pharmacists.	• Collaborate with supportive healthcare providers and religious leaders.

Perceived barriers were identified during the exploratory phase and presented to stakeholders during the consultation to inform discussion and solution generation. Short-term solutions represent stakeholder-proposed approaches considered suitable for pilot implementation, whereas long-term solutions represent broader system-level, policy, or regulatory changes that extend beyond the scope of the pilot and may require additional resources, stakeholder engagement, or policy reform. The consultation did not include formal prioritization or ranking of barriers or solutions.

√ Proposed solution incorporated into the pilot pharmacy-led PrEP service model; proposed solutions without this symbol were not incorporated into the pilot and may inform future implementation planning, service refinement, or broader system-level changes.

Abbreviations: CHW, community health worker; TDF/FTC, tenofovir disoproxil fumarate/emtricitabine; MOH, Ministry of Health Malaysia; NGO, non-governmental organization; MASHM, Malaysian Society for HIV Medicine; SOGIE, Sexual Orientation, Gender Identity, and Expression.

### Proposed short-term solutions

Short-term solutions focused on improving service visibility, safeguarding privacy and confidentiality, strengthening pharmacist capacity, simplifying workflows, and ensuring adherence to national guidelines. For demand generation, stakeholders recommended targeted digital promotion, collaboration with NGOs and telemedicine providers, and integration with existing community referral networks serving key populations. Additional strategies included listing participating pharmacies on the My PrEP Locator [31], implementing digital appointment systems, and supporting outreach through community health workers and peer networks. To support safe and practical service delivery, stakeholders emphasized the importance of private consultation and testing spaces, restricted access to client information, integration of HIV self-testing (HIVST) using Medical Device Authority-approved kits, and clear referral pathways with physicians, laboratories, and community health workers to support confirmatory testing, psychosocial care, and continuity of care. To simplify testing requirements during the pilot phase, baseline kidney function testing was recommended, while sexually transmitted infections (STIs) and hepatitis screening remained optional depending on affordability and client willingness.

Operational recommendations included implementing services in pharmacies with at least two full-time pharmacists, scheduling appointments to manage workload, strengthening pharmacist preparedness through comprehensive PrEP training and reference guides, promoting stigma-sensitive and client-centered care through Sexual Orientation, Gender Identity and Expression (SOGIE) sensitization training, and ensuring accurate electronic documentation and dispensing only with valid physician prescriptions. Medication rebates were introduced during the pilot phase to reduce financial barriers. Although pre-visit online risk assessments, online consultations during peak hours, and tele-pharmacy with medication delivery options were discussed, stakeholders considered an in-person service model more practical during the pilot phase. In addition, placing community health workers at pharmacies to support testing and enrolment was considered operationally challenging because of NGO workforce constraints and limited pharmacy space.

### Proposed long-term solutions

Long-term solutions focused on improving affordability, workforce capacity, service quality, and policy support. Strategies to improve affordability and sustainability included public-private partnerships with MOH to explore reimbursement mechanisms, negotiating lower PrEP prices, expanding insurer coverage and MOH reimbursement for PrEP and HIV test kits, and pursuing tax relief.

To strengthen workforce capacity and service quality, stakeholders recommended accredited PrEP training, online courses, ongoing mentorship, integration of HIV prevention training into undergraduate pharmacy curricula, standardized implementation toolkits, monitoring systems, and clear HIV self-testing guidelines. Partnerships with NGOs or MOH to provide free or subsidized testing were also proposed.

Policy-related strategies included exploring pharmacist prescribing as part of task-sharing efforts, which may require regulatory changes such as down-regulating oral TDF/FTC under the Poisons Act, and establishing a unique client identification system to support continuity of care across pharmacies. To strengthen public trust and accountability, stakeholders proposed a community-endorsed PrEP pharmacy rating system. Long-term efforts to address stigma and enhance acceptability may include sustained collaboration with healthcare providers, community leaders, and religious leaders to support ethical, inclusive, and client-centered PrEP service delivery.

### Pilot study procedures, care pathway, workflow, schedule of activities and assessment tools

Stakeholder feedback informed refinement of the pilot study procedures, care pathway, workflow, schedule of activities, and assessment tools to improve practicality, safety, inclusiveness, and alignment with the Malaysian healthcare and regulatory context. Detailed feedback is summarized in S5 Appendix. The pilot study design was shaped by Malaysia-specific regulatory, policy, and health system constraints affecting the implementation of pharmacy-led PrEP service delivery, including prescribing authority, HIV testing regulations, laboratory access, and financing structures (S6 Appendix).

Key modifications to the pilot procedures included refinements to eligibility criteria, follow-up schedules, referral guidance, management of reactive HIV self-test results, and assessment tools. Eligibility criteria focused on individuals aged 18–49 residing in the Klang Valley who were at lower risk of kidney impairment and willing to use PrEP and undergo laboratory testing at their own expense. Individuals aged 50 years and above or those with kidney-related comorbidities were excluded because they may require closer renal monitoring that may not be feasible in community pharmacy settings without on-site laboratory services [8]. Individuals with comorbidities, pregnancy or breastfeeding, or reported allergies to PrEP medications were also excluded and referred to physicians for further evaluation and management.

Stakeholders also recommended adjustments to improve inclusiveness and clinical interpretation. Pharmacist-assisted consent was permitted for clients who do not understand English or Malay. Stakeholders also discussed the eligibility of individuals using creatine supplements, as creatine may elevate serum creatinine and lower estimated glomerular filtration rate (eGFR) without indicating true renal impairment [32]. Pharmacists were advised to counsel such clients on kidney function monitoring and consider repeat testing when appropriate.

Follow-up schedules were refined for different PrEP user groups. New users and restarting users, defined as individuals who had not used PrEP within the previous six months [33], were scheduled for a follow-up visit one month after initiation to account for the HIV self-test window period, assess side effects, and reinforce adherence, followed by routine visits every three months thereafter [33]. For continuing users, the month-one visit could be omitted if a negative HIV test had been documented or self-reported within the preceding three months.

Stakeholders also strengthened referral guidance for pharmacists. Pharmacists were advised to distinguish between true allergic reactions and common side effects before referring clients to physicians while clients reporting missed PrEP doses with potential HIV exposure should be referred for post-exposure prophylaxis (PEP) evaluation according to established criteria.

For reactive HIV self-test results, stakeholders emphasized that HIV self-testing should be communicated to clients as a screening tool rather than a diagnostic test. Clients with reactive HIV self-test results should be referred for confirmatory testing at community-friendly PrEP clinics or through community health workers, with notification to the study team to facilitate follow-up and linkage to care. Stakeholders also clarified that pharmacists are not responsible for HIV case notification to the Ministry of Health, as only confirmed physician diagnoses are reportable under national guidelines [34].

**Fig 1:**
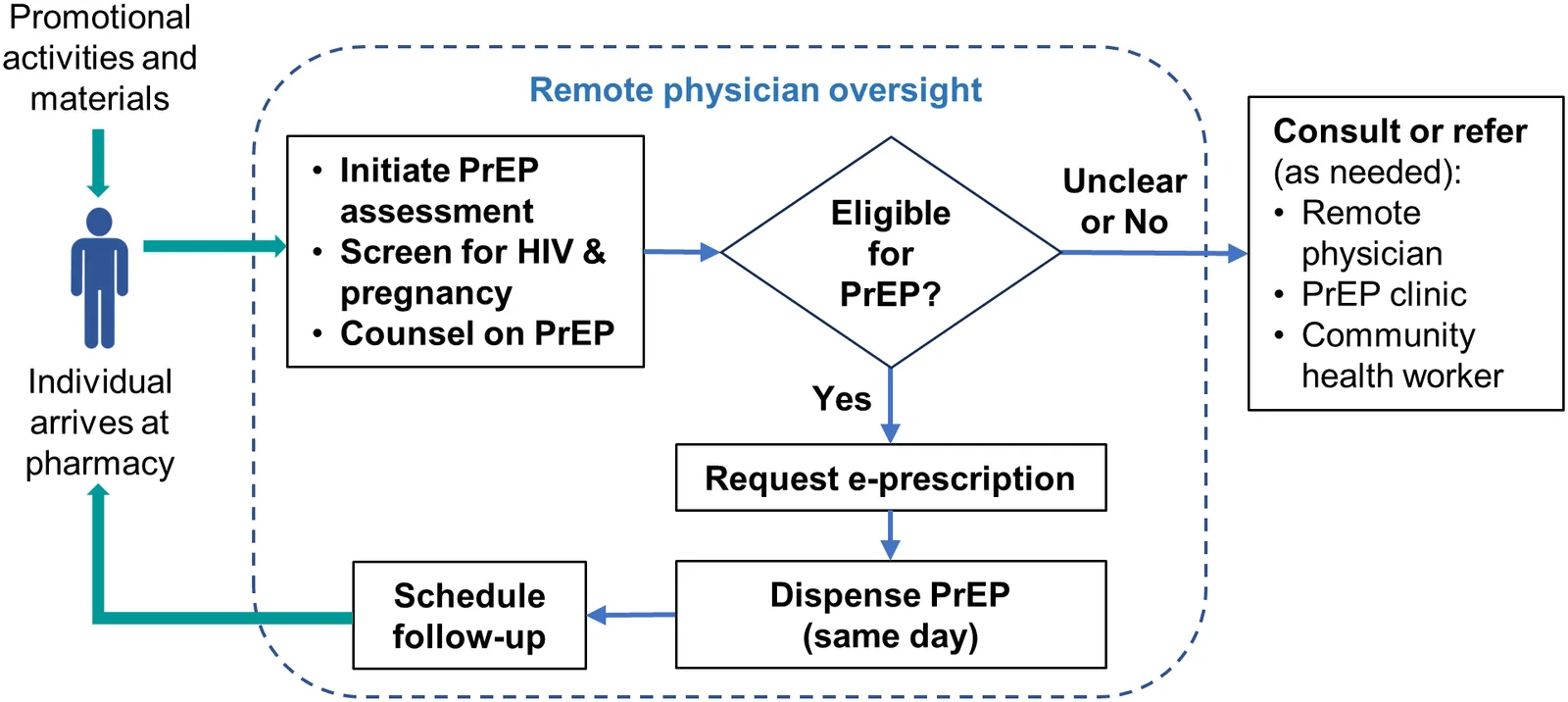
Proposed care pathway for the pharmacy-led PrEP service pilot study in Malaysia. Adapted from Ortblad KF, Mogere P, Roche S, et al. Design of a care pathway for pharmacy-based PrEP delivery in Kenya: results from a collaborative stakeholder consultation. BMC Health Serv Res. 2020;20:1034. https://doi.org/10.1186/s12913-020-05898-9. Licensed under CC BY 4.0.

**Fig 2:**
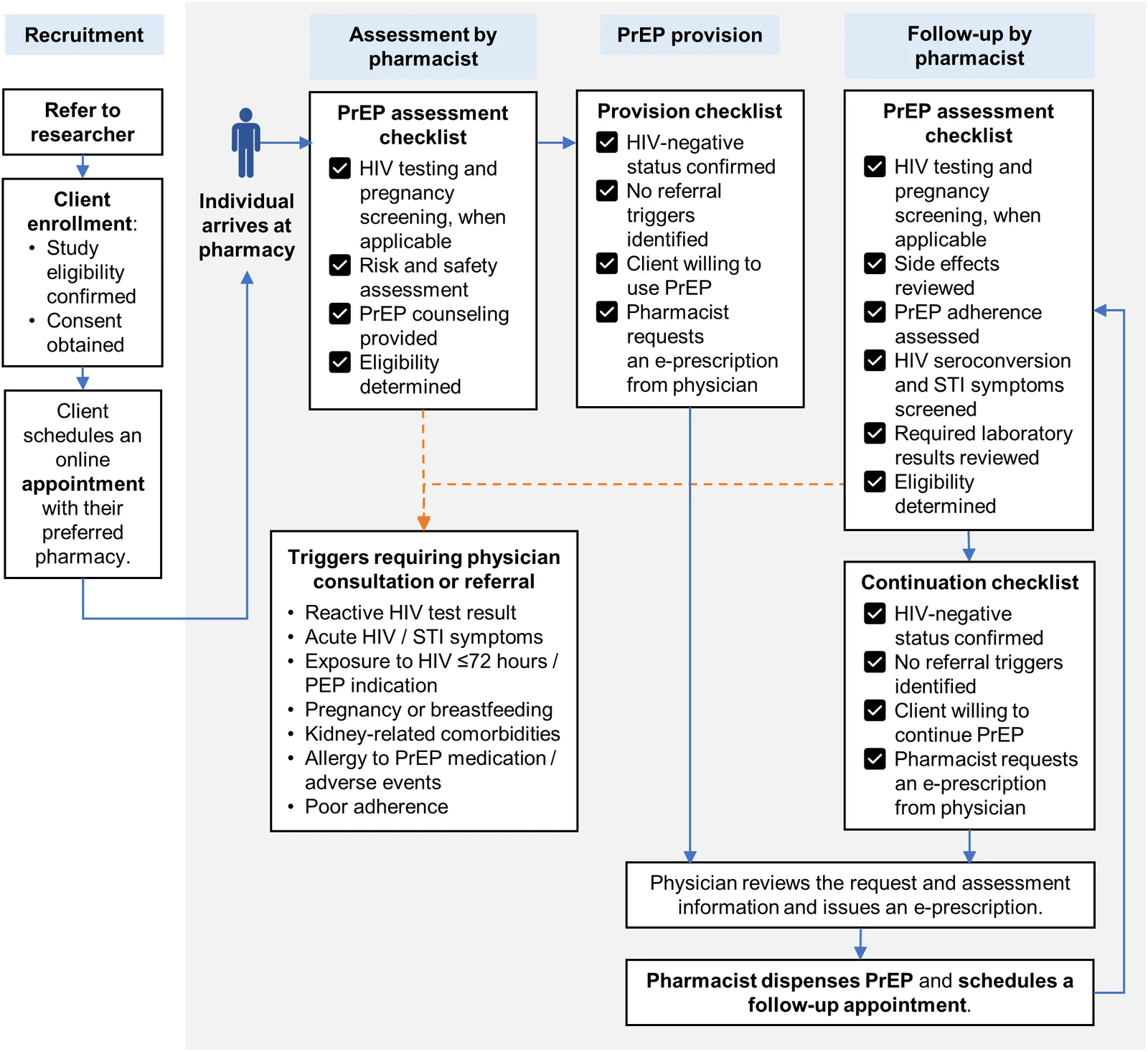
Proposed workflow for the pharmacy-led PrEP service pilot study in Malaysia.

Stakeholders reviewed and supported the proposed care pathway (Fig 1), workflow (Fig 2), schedule of activities (S3 Appendix), and assessment tools (S4 Appendix), and considered these materials suitable for pilot testing. The care pathway integrates remote physician oversight and referral procedures for clients with unclear eligibility or clinical concerns, while the schedule of activities outlines the timing of assessments, laboratory testing, follow-up visits, and monitoring throughout the pilot study. Supporting these processes, the workflow outlines pharmacy-led assessment, counseling, HIV self-testing, prescribing, dispensing, and follow-up, supported by a digital checklist-based assessment tool integrated into the telemedicine platform to standardize documentation and facilitate communication between pharmacists and prescribing physicians.

Stakeholders also refined the assessment tools to improve usability and clinical relevance. Modifications included adding a preferred name field to support respectful engagement with key populations, clarifying referral criteria for possible HIV seroconversion, and incorporating STI symptom screening at each visit. Adherence monitoring was strengthened through combined qualitative and percentage-based assessments, with counseling recommended for initial low adherence and physician referral considered for persistent low adherence with ongoing HIV risk.

### Training needs and competencies

Suggested training topics covered PrEP fundamentals, including pharmacology, effectiveness, dosing, HIV testing, relevant laboratory tests, counseling, and dispensing. Stakeholders also emphasized foundational knowledge of HIV and STIs, together with skills such as sexual history-taking, identifying potential PrEP candidates, assessing HIV risk behaviors, and determining eligibility for initiation and continuation. Additional topics included risk reduction strategies, referral pathways and linkage to care, and post-test counseling. To support client-centered care, training should incorporate SOGIE, stigma and discrimination awareness, communication skills, and understanding community-specific contexts. Scenario-based learning was recommended to prepare pharmacists for situations such as managing reactive HIV test results and addressing adherence challenges.

Stakeholders also identified key competencies required for effective PrEP service delivery. Knowledge competencies included understanding HIV, STIs, and risk behaviors, conducting HIV risk assessments, and recognizing when and where to refer clients, including to NGOs or clinics. Pharmacists should be able to manage reactive HIV test results and support linkage to confirmatory testing and care. Behavioral competencies emphasized professionalism, empathy, non-judgmental attitudes, emotional intelligence, and strong communication skills. Pharmacists were expected to prioritize client privacy, use person-centered language, and demonstrate sensitivity to SOGIE and the needs of key populations.

### Study site selection and site preparation requirements

Stakeholders discussed site selection and preparation requirements to ensure accessibility and operational readiness. Site selection criteria included location within Klang Valley, availability of a private consultation room, at least two full-time pharmacists, willingness to complete PrEP training, subscription to the DOC2US^®^ telemedicine platform [35], and proximity to nearby PrEP clinics.

Alpro Pharmacy, a private pharmacy chain with more than 300 outlets across Malaysia, served as the implementation partner [36]. Outlets in the Klang Valley were selected based on the distribution of existing PrEP providers identified through the My PrEP Locator [31]. Remote physician oversight and prescribing via the DOC2US^®^ platform enabled electronic prescriptions and same-day PrEP provision, necessitating platform subscription. A partnering PrEP clinic within a 10 km radius was required to support referrals for confirmatory testing, laboratory services, STI screening, and PEP. Six pharmacies meeting these criteria were selected in Petaling Jaya, Bandar Puteri Puchong, Bukit Serdang, Bandar Sri Permaisuri, Sungai Besi, and Cyberjaya.

Site preparation requirements aligned with WHO recommendations [37] and included availability of HIV self-test kits, PrEP medications, client education materials, and integration of the assessment tool into the telemedicine platform. Trained pharmacists, remote physicians, and established referral networks with nearby clinics and community health workers were also required. Sites were listed on the My PrEP Locator to improve service visibility [31].

### Promotional activities and materials

Stakeholders emphasized targeted promotional strategies to increase awareness and demand for pharmacy-led PrEP services among key populations. Digital promotion through websites, social media, and telemedicine platforms was recommended, alongside collaboration with NGOs, pharmacies, and community influencers to support outreach. Pharmacies were encouraged to display PrEP service stickers, and rebate vouchers were introduced through partnerships with suppliers and pharmacy providers to reduce medication costs during the study period.

Stakeholders reviewed and endorsed recruitment advertisements used for outreach. Educational materials for individuals at risk of HIV acquisition were developed, including PrEP leaflets describing indications, dosing (daily or on-demand), potential side effects, and access to services. Translation into Malay was considered important for accessibility. The materials also incorporated feedback from key-population representatives and were reviewed by the MOH Pharmaceutical Services Program for regulatory compliance.

### Key implementation considerations

**Fig 3:**
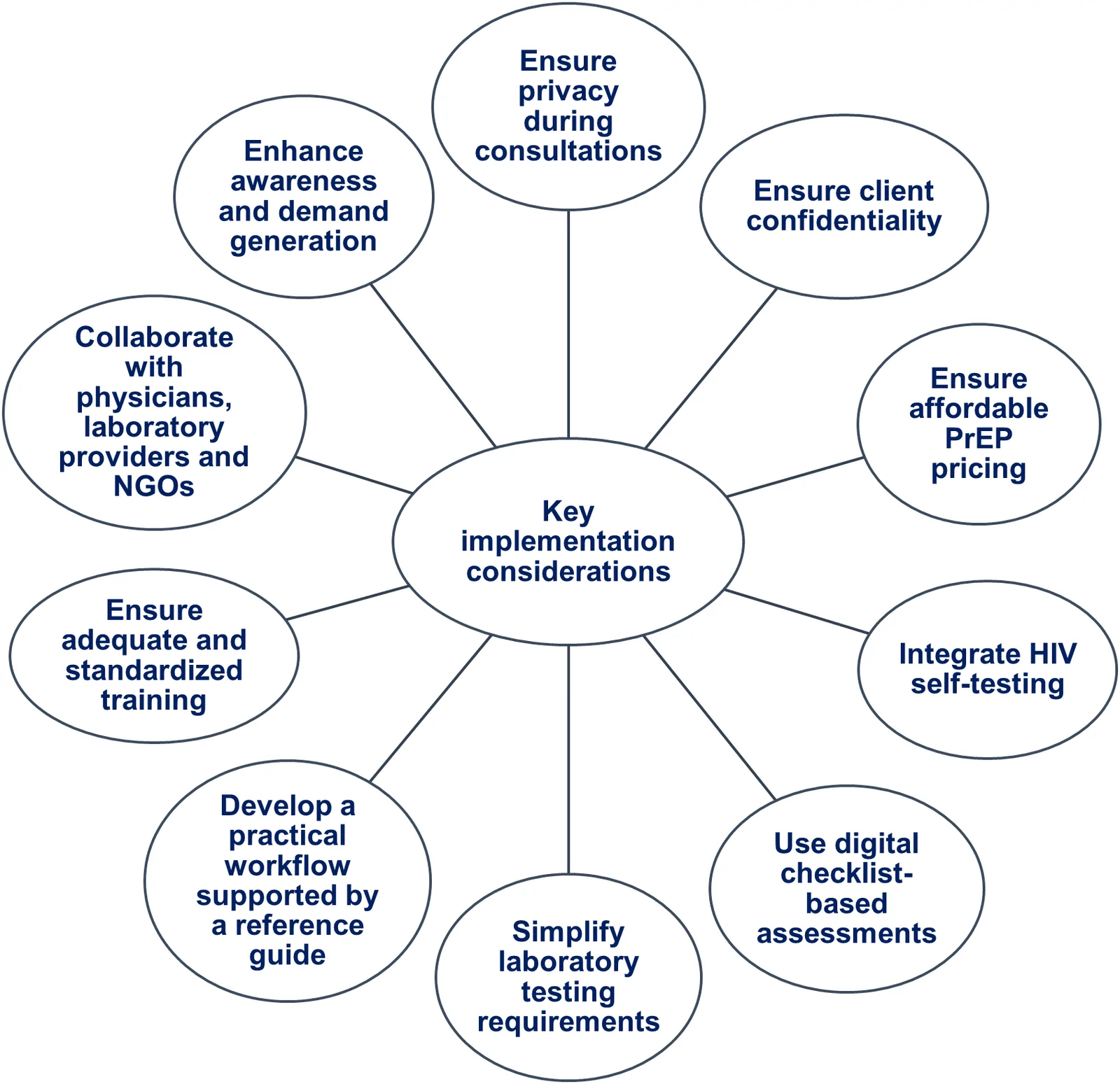
Key implementation considerations from stakeholder inputs across the consultation activities.

Through descriptive synthesis, the research team organized recurring stakeholder inputs across the consultation activities into ten researcher-derived implementation considerations (Fig 3). These considerations were used as researcher-derived descriptive categories to summarize recurrent implementation issues and recommendations; they were not formally validated qualitative themes or stakeholder-ranked priorities. Several implementation considerations, including awareness and demand generation, privacy and confidentiality, affordability, HIV self-testing, pharmacist training, and collaboration with physicians, laboratory providers, and NGOs, were consistently identified across discussions on proposed short-term solutions. Other implementation considerations, including simplifying laboratory testing requirements, using digital checklist-based assessments, and developing practical workflows supported by reference guides, emerged primarily through review and discussion of the proposed pilot procedures, care pathway, workflow, and assessment tools.

No formal prioritization, scoring or thematic analysis was undertaken. The researcher-derived implementation considerations informed refinement of the pilot implementation plan, while recommendations requiring broader regulatory, policy or financing changes were retained for future service adaptation and scale-up.

## Discussion

This consultation translated stakeholder input into a context-specific implementation plan for pharmacy-led PrEP delivery in Malaysia. Participating stakeholders repeatedly identified the need to protect privacy and confidentiality, address affordability, integrate HIV self-testing, standardize pharmacist training, coordinate physician-supported prescribing, and establish reliable laboratory and community referral pathways. To our knowledge, this is the first reported Malaysian stakeholder consultation focused specifically on implementing pharmacy-led PrEP. Consistent with experiences from Kenya and Nigeria, stakeholders emphasized the need for pragmatic service delivery, streamlined workflows, and collaboration across healthcare providers and community organizations. However, Malaysia’s regulatory and health system contexts, including prescribing restrictions, limitations on HIV testing in private pharmacies, reliance on private-sector financing, and the criminalization of key populations, present implementation challenges that require careful contextual adaptation.

The consultation identified practical short-term solutions that could be integrated into routine service delivery, while reserving more resource-intensive strategies for future scale-up. Stakeholder input informed revisions to the pilot study procedures, assessment tools, and implementation processes to improve alignment with stakeholder-identified operational needs and preferences.

Demand generation emerged as a key consideration, consistent with studies from Kenya and Nigeria, where low awareness of pharmacy-based PrEP services limited uptake [22,28]. In the Malaysian context, persistent stigma and limited public awareness of PrEP further constrain service uptake. Addressing these barriers will require community-engaged strategies, including partnerships with NGOs and targeted digital outreach, to improve visibility and acceptability of pharmacy-led PrEP services.

Ensuring privacy and confidentiality was identified as critical for implementation, similar to findings from Nigeria and global reviews of pharmacy-led PrEP services [22,38,39]. Within Malaysia, concerns around stigma and disclosure may heighten the need for discreet and confidential service delivery. Ensuring privacy through private consultation rooms and confidentiality through restricted access to client information, limited to pharmacists and physicians directly involved in care, will therefore be essential to support service uptake.

Because the service operates within Malaysia’s predominantly out-of-pocket private healthcare sector, affordability is likely to influence service uptake and is therefore an important implementation consideration. In addition, many professional community pharmacy services in Malaysia are provided without a formal remuneration mechanism, which may raise concerns regarding the financial sustainability and opportunity costs of delivering additional clinical services [40]. Evidence from Kenya suggests that willingness to pay for pharmacy-delivered PrEP is closely linked to local purchasing power [14]. A Malaysian study published in 2017 found that only 35.6% of MSM were willing to pay for PrEP out-of-pocket, with over half preferring a monthly cost below RM100 [41].

At the time of the pilot, PrEP medication cost RM97 per month and an HIV self-test kit cost RM60 when required. Thus, RM157 represented the medication-plus-HIV self-testing cost in a month when both were purchased; it was not the complete cost of PrEP initiation or a recurring monthly total. Baseline renal-function testing was paid separately and was not included because costs varied by testing provider and testing was not repeated at every visit. No pharmacy consultation or service fee was charged, while telemedicine costs were absorbed by participating pharmacies through their existing subscription arrangements. During the pilot, medication rebates reduced the PrEP medication cost to RM65 per month, and HIVST was provided at no charge. As the willingness-to-pay estimates were based on survey data collected in 2016, direct comparison with these costs should be interpreted with caution, given potential changes in economic conditions, PrEP awareness, and healthcare financing. Sustainable financing mechanisms and cost-sensitive implementation strategies will be essential to ensure affordability, equitable access, and long-term scalability beyond the subsidized pilot.

Stakeholders supported integrating HIVST to simplify service delivery and streamline PrEP initiation and continuation, in line with WHO recommendations [8]. At the time of consultation, provider-assisted HIVST was the only form of HIV testing permitted in private pharmacies in Malaysia; national HIVST guidelines were subsequently introduced in December 2023 [25,42]. Similar constraints have been reported in Kenya, where pharmacies may assist clients with HIVST but are not permitted to perform rapid diagnostic testing [28], whereas Nigeria has recently expanded regulations to allow pharmacy-based rapid HIV testing [22]. These differences highlight how regulatory frameworks shape the extent to which HIV testing can be decentralized to pharmacies. In this context, assisted HIVST was viewed by stakeholders as a practical interim approach that enables private, client-centered HIV screening while maintaining regulatory compliance.

Stakeholders recommended simplifying laboratory requirements to reduce barriers to PrEP access. This is in line with WHO guidance, which supports more flexible and risk-based laboratory monitoring approaches [8]. Similar approaches have been applied in Kenya, where hepatitis B, hepatitis C, and kidney function tests were not routinely performed and their absence did not prevent PrEP initiation [14]. In Malaysia, where access to laboratory services may be limited within pharmacy settings or add to out-of-pocket costs, extensive testing requirements may create additional barriers to PrEP uptake. Focusing on essential tests such as HIV screening while deferring non-critical investigations may improve access and reduce costs.

Digital tools were identified by stakeholders as important for standardizing processes, improving workflow efficiency, and enhancing documentation. Digitalized checklist-based assessment tools support consistent PrEP eligibility assessment, facilitate electronic prescribing, and ensure adherence to safety and referral criteria. Similar structured checklists have been used in Kenya to support pharmacy providers in determining PrEP eligibility and identifying cases requiring medical referral [14]. Given that pharmacists in Malaysia have limited prior experience with PrEP provision, such tools may support consistent clinical decision-making, improve documentation, and promote safe service delivery.

Stakeholders underscored the need for clear workflows and effective coordination across the healthcare system. Differences in regulatory frameworks across settings, including pharmacist prescribing authority in the United States and checklist-based dispensing models in Kenya [10–14], highlight how pharmacy-led PrEP delivery must be adapted to local contexts. In Malaysia, prescribing restrictions necessitate continued physician involvement, which may introduce additional coordination steps but also ensures clinical oversight. Stakeholders considered streamlined workflows and effective collaboration between pharmacists and physicians important for maintaining timely access while ensuring regulatory compliance and patient safety.

Structured guidance was considered essential by stakeholders to support consistent PrEP delivery in pharmacy settings. Lack of clear guidance has been reported as a barrier to identifying and counseling individuals at risk [38]. As pharmacists in Malaysia may have limited prior experience with PrEP provision, providing clear, evidence-based reference tools may support consistent clinical decision-making, improve pharmacist confidence, and enhance service quality.

Stakeholders viewed referral networks and partnerships with NGOs, prescribers, and laboratory providers as critical for supporting outreach, testing, and continuity of care in pharmacy-led PrEP services. Evidence from other studies similarly highlights the role of integrated referral pathways in enabling effective PrEP delivery [15,38]. In Malaysia, where pharmacies typically lack on-site laboratory services and are not fully integrated within the broader healthcare system, such partnerships are essential to ensure timely testing, linkage to care, and management of complex cases. Strengthening referral pathways may therefore be critical to ensuring safe and efficient PrEP service delivery.

Stakeholders highlighted the need for structured training to equip pharmacists with competencies beyond routine dispensing, including PrEP assessment, HIVST support, counseling, and referral management. Adequate training has been identified as essential for the successful implementation of pharmacy-led PrEP services, as improved PrEP knowledge enhances dispensing practices and counseling confidence [43,44]. At the time of consultation, no pharmacist-specific PrEP training was available locally; only an online PrEP and PEP course for general practitioners was available [45]. The stakeholder recommendations subsequently informed the development of a pharmacist PrEP training program comprising self-paced online learning followed by an in-person workshop to support pilot implementation. The development and evaluation of the online training component have been reported separately [30]. Structured, competency-based training programs that combine online learning with practical components are likely to be essential to support consistent and scalable implementation. However, the resources required to train and certify pharmacists at scale remain an important consideration for future implementation planning.

Accessibility and operational readiness were considered by stakeholders to be important site selection criteria for pharmacy-led PrEP services. In line with WHO guidance [37], site readiness depends on key enabling components, including private consultation spaces, trained pharmacists, and linkages with telemedicine and referral networks. In the Malaysian context, where variability in pharmacy infrastructure and service capacity may affect implementation, careful site selection is critical to support high-quality service delivery. Emphasizing operational readiness may support smoother implementation and reduce workflow disruptions during service delivery.

Consistent with stakeholder consultations conducted in Kenya and Nigeria, this consultation identified privacy and confidentiality, affordability, workforce training, workflow design, and collaboration between service providers as important considerations for pharmacy-led PrEP implementation [22,28]. However, several recommendations reflected the Malaysian regulatory and healthcare context, particularly the need for physician prescribing partnerships, integration of telemedicine services, and the use of assisted HIVST within existing regulatory requirements. These findings highlight both the transferability of key implementation considerations across settings and the importance of adapting pharmacy-led PrEP models to local service delivery and regulatory contexts.

The proposed implementation model may also have relevance for other middle-income countries in Southeast Asia seeking to expand PrEP access through private-sector and community-based service delivery. Components such as pharmacist training, telemedicine-supported prescribing, HIVST, structured referral pathways, and partnerships with community organizations may be adaptable across settings. However, transferability will depend on country-specific regulatory frameworks governing pharmacist scope of practice, HIV testing, and PrEP prescribing, as well as healthcare financing, pharmacy infrastructure, and the availability of laboratory and referral services. The Malaysian model should therefore be viewed as a contextually adaptable implementation approach rather than a directly transferable service model.

Since the consultation was conducted in 2023, the MOH published national guidelines in 2025 to support implementation of the national PrEP program within public healthcare services [46]. However, guidance for private-sector PrEP delivery models, including pharmacy-led and telemedicine-supported approaches, remains limited. Recent evidence suggests growing interest among Malaysian key populations in alternative PrEP modalities, including long-acting oral and injectable PrEP [47]. In addition, implementation research on pharmacy-based delivery of long-acting injectable antiretrovirals has identified similar considerations related to privacy, training, workflow design, and interprofessional collaboration, suggesting that several considerations identified through this consultation are likely to remain important as PrEP delivery evolves [48]. Future research should explore how pharmacy-led service delivery can be adapted to support different PrEP modalities while maintaining accessibility, affordability, and person-centered care. Nevertheless, these implementation considerations reflect the policy, regulatory, and service delivery context at the time of the consultation. Their relative relevance may evolve as national HIV policies, PrEP delivery models, prescribing arrangements, and regulatory frameworks continue to develop. Periodic stakeholder reassessment will be needed to ensure that the service delivery model remains responsive to changing healthcare needs and implementation contexts.

This study had several limitations. First, the private pharmacy and telemedicine stakeholders represented a selective group already committed to participating in the pilot study, which may have limited the diversity of perspectives and influenced recommendations toward approaches considered practical within the participating organizations. Stakeholders with more conservative views on pharmacist roles, differing regulatory perspectives, or greater reservations about pharmacy-led PrEP implementation may have been underrepresented. Nevertheless, the consultation was intended to inform implementation within the participating pilot sites, which were supported by an existing telemedicine and physician-prescribing model. As such, the inclusion of stakeholders involved in these service delivery settings provided practical insights into implementation challenges and operational requirements relevant to the pilot.

Second, although the consultation included community-based organization representatives and community health workers from key populations, participation by a broader range of individual end-users was limited. Consequently, perspectives on privacy, stigma, acceptability, and service preferences should be interpreted alongside findings from the preceding exploratory phase, which directly captured the views of key populations.

Third, the consultation was conducted to support implementation of an urban, private-sector service model in the Klang Valley. Therefore, the findings may not fully reflect implementation challenges, resource constraints, or service delivery considerations in rural settings, regions with more limited healthcare infrastructure, or pharmacy settings without established telemedicine and physician-prescribing partnerships or comparable operational resources.

Fourth, representatives from the HIV/STI/Hepatitis C Sector of the MOH Disease Control Division and the MOH Pharmacy Program were not included in their official programmatic or regulatory capacities, as the consultation focused on implementation planning for a pilot of a private-sector pharmacy-led PrEP service model operating within existing regulatory frameworks. Their participation could have provided additional perspectives on regulatory alignment and national scale-up pathways, particularly for the longer-term solutions identified during the consultation. Nonetheless, participation from diverse public- and private-sector stakeholders provided valuable insights that strengthened the implementation plan.

Fifth, the consultation did not include formal prioritization of barriers or implementation strategies. Although stakeholders differentiated between short- and long-term solutions, this approach did not determine which barriers or strategies were considered most critical. In contrast, the Nigeria stakeholder consultation applied the Nominal Group Technique (NGT) to prioritize implementation strategies systematically [22]. While this pragmatic approach enabled identification of recurring implementation considerations and refinement of the service model, it limited the ability to systematically compare the relative importance of implementation barriers and strategies. Future consultations could incorporate structured prioritization methods, such as the NGT, to establish clearer implementation priorities.

Sixth, stakeholder inputs were synthesized descriptively and were not subjected to formal qualitative coding. While this pragmatic approach supported refinement of the service model and identification of recurring implementation considerations, formal coding may have provided a more systematic categorization of stakeholder inputs and greater transparency in how individual inputs contributed to the final implementation considerations.

Lastly, the pilot study focused exclusively on in-person pharmacy visits. A discrete choice experiment among Malaysian MSM found preferences for same-day testing with online monitoring (63.1%), followed by models involving online consultations, mail-order PrEP, and online monitoring (16.2%), and self-screening via online questionnaire (11.3%) [49]. Hybrid models involving tele-pharmacy and home delivery of HIVST kits or PrEP medications were not implemented due to cost and operational constraints. However, these approaches may reduce stigma and increase convenience and should be explored in future iterations of pharmacy-led PrEP service delivery.

Overall, this consultation identified key implementation considerations for pharmacy-led PrEP service delivery in Malaysia. These findings highlight the importance of adapting pharmacy-led PrEP models to local regulatory, financing, and health system contexts. The insights generated provide a practical foundation for pilot implementation and may inform future adaptation and scale-up of pharmacy-led PrEP services in similar urban private-sector settings. Prospective evaluation of the resulting service model is needed to assess reach, adoption, fidelity, acceptability, cost, PrEP initiation, and continuity of care and to determine whether the stakeholder-informed adaptations improve implementation in practice.

## Conclusions

This study describes the stakeholder-informed refinement of a pharmacy-led PrEP service delivery model for pilot implementation in an urban private-sector setting in Malaysia. Key recommendations focused on enhancing awareness and demand generation, ensuring privacy, confidentiality, and affordability, integrating HIV self-testing, using digital checklist-based assessments, simplifying laboratory testing requirements, developing a practical workflow supported by a reference guide, strengthening pharmacist training, and fostering cross-sector collaboration. These findings provide practical guidance for pilot implementation and may inform future adaptation of pharmacy-led PrEP services in similar urban, private-sector settings where physician prescribing, assisted HIV self-testing, laboratory access, and out-of-pocket financing shape service delivery.

## Supporting information

S1 Appendix Preliminary findings from exploration phase: Perceived barriers and facilitators regarding the pharmacy-led PrEP service from the perspectives of key populations, community pharmacists and PrEP prescribers. (PDF)

S2 Appendix Worksheet. (PDF)

S3 Appendix Schedule of activities. (PDF)

S4 Appendix Assessment tools for pharmacy-led PrEP service initiation and follow-up. (PDF)

S5 Appendix Stakeholder feedback on pilot study procedures, care pathway, workflow, schedule of activities, and assessment tools. (PDF)

S6 Appendix Malaysia-specific implementation considerations and adaptations for pharmacy-led PrEP service delivery. (PDF)
