# Supplementary material for: Stakeholder-informed pre-implementation refinement of a pharmacy-led pre-exposure prophylaxis (PrEP) service delivery model in Malaysia
Source: PLoS One. 2026 Sep 8;21(9):e0357920. doi: 10.1371/journal.pone.0357920 (PMC13552737; doi:10.1371/journal.pone.0357920)
Supplement: Supplementary file 1 [file pone.0357920.s001.pdf]

S1 Appendix. Preliminary findings from exploration phase: Perceived barriers and facilitators regarding the pharmacy-led PrEP service from the perspectives of key populations, community pharmacists and PrEP prescribers.

Table S1.1 Perceived barriers regarding pharmacy-led PrEP service from the perspectives of key populations, community pharmacists and PrEP prescribers.

<table>
<tr><th>All</th><th>Key populations</th><th>Community pharmacists</th><th>PrEP prescribers</th></tr>
<tr>
<td rowspan="3">• Poor awareness about PrEP
• Poor awareness about pharmacy-led PrEP service
• Concerns about privacy and confidentiality
• Stigma and discrimination
• High costs / affordability
• Concerns about pharmacists' professionalism and capabilities
• Lack of laboratory services</td>
<td>• Unfamiliarity / uncertainty regarding a new service</td>
<td>• Busy environment
• Time constraints
• Lack of manpower
• Increased workload
• Concerns about documentation of client record
• Profitability / opportunity cost
• Lack of demand
• Uncertainty about client disclosure / clients withholding information
• Concerns about potential loss to follow up
• Discomfort among staff and customers
• Personal / religious beliefs</td>
<td>• Accuracy of oral-fluid HIVST kits
• Concerns about kidney function monitoring
• Concerns about the ability to support clients with reactive test results
• Concerns about the ability to provide effective counselling
• Concerns about potential non-adherence to guidelines / pilot study procedures</td>
</tr>
<tr>
<td colspan="2">• Concerns about PrEP use (side effects, kidney impairment)</td>
<td></td>
</tr>
<tr>
<td></td>
<td colspan="2">• Lack of knowledge / skills / experience
• Insufficient training
• Unwillingness of pharmacists to provide service</td>
</tr>
</table>

Table S1.2 Perceived facilitators regarding pharmacy-led PrEP service from the perspectives of key populations, community pharmacists and PrEP prescribers.

<table>
<tr><th>All groups</th><th>Key populations</th><th>Community pharmacists</th><th>PrEP prescribers</th></tr>
<tr>
<td rowspan="3">Increased awareness and demand for PrEP
Effective promotion of service
Availability of private room / space
Assurance of confidentiality
Affordable pricing
Adequate training
Effective PrEP counselling
Non-judgmental service
Friendly service
Person-centred care
Quick / time-saving service
Proper follow-up and monitoring
Physician oversight
Proactive follow-up and reminders
Referral procedures and resources
Collaboration with relevant stakeholders for effective referral
Regulatory compliance
Educational materials</td>
<td>Adherence to local guidelines / pilot study procedures
Same-day PrEP
User-friendly appointment scheduling system</td>
<td>Availability of PrEP medication
Availability of reference guide
Financial sustainability</td>
<td>Adherence to local guidelines / pilot study procedures
Same-day PrEP
User-friendly appointment scheduling system
Digitalized checklist-based assessment tool
Utilization of HIV self-testing
Simplified kidney function monitoring
Incentives
Adequate shared information for prescribing decision
Adherence counselling and monitoring</td>
</tr>
<tr>
<td colspan="2">Accredited training for pharmacists
Adequate knowledge about HIV and PrEP
Familiarity with key populations' needs
Good communication skills
Insurance coverage
Increased staffing
Comfortable environment</td>
<td></td>
</tr>
<tr>
<td></td>
<td colspan="2">Clear guidelines for service delivery
Proper documentation of client records
Willingness of pharmacists to provide service
Continuous medical education</td>
</tr>
</table>
