# Supplementary material for: Stakeholder-informed pre-implementation refinement of a pharmacy-led pre-exposure prophylaxis (PrEP) service delivery model in Malaysia
Source: PLoS One. 2026 Sep 8;21(9):e0357920. doi: 10.1371/journal.pone.0357920 (PMC13552737; doi:10.1371/journal.pone.0357920)
Supplement: Supplementary file 2 [file pone.0357920.s002.pdf]

S2 Appendix. Worksheet.

Core components	Barriers	Potential solutions	
		Short-term	Long-term
**Promotional activities & materials**	● Poor awareness about PrEP<br>● Poor awareness about the pharmacy-led PrEP service<br>● Lack of demand<br>● Unfamiliarity / uncertainty regarding a new service		
**HIV testing**	● Concerns about privacy and confidentiality<br>● High costs / affordability<br>● Accuracy of oral fluid HIV self-test kits<br>● Ability to support clients with reactive test results		
**Assessment and counseling**	● Concerns about privacy and confidentiality<br>● Concerns about pharmacists' professionalism and capabilities<br>● Ability to provide effective counselling<br>● Lack of knowledge / skills / experience<br>● Insufficient training<br>● Time constraints<br>● Profitability / opportunity cost<br>● Increased workload<br>● Lack of manpower<br>● Uncertainty about client disclosure / clients withholding information<br>● Lack of laboratory services<br>● Concerns about kidney function monitoring<br>● Adherence to guidelines / pilot study procedures<br>● Concerns about documentation of client record		
**Prescribing by physician**	● Adherence to guidelines / pilot study procedures<br>● Concerns about documentation of client record		
**Dispensing**	● High costs / affordability<br>● Profitability / opportunity cost<br>● Adherence to guidelines / pilot study procedures		
**Follow-up**	● Concerns about pharmacists' professionalism and capabilities<br>● Ability to support clients with reactive test results<br>● Ability to provide effective counselling<br>● Concerns about PrEP use (side effects, kidney impairment)<br>● Concerns about documentation of client record<br>● Concerns about potential loss to follow up		
**Oversight & Referrals**	● Concerns about pharmacists' professionalism and capabilities<br>● Ability to support clients with reactive test results<br>● Ability to provide effective counselling<br>● Concerns about documentation of client record<br>● Adherence to guidelines / pilot study procedures		
**Others**	● Stigma and discrimination<br>● Personal / religious beliefs<br>● Unwillingness of pharmacists to provide service<br>● Discomfort among staff and customers		
