# Supplementary material for: Stakeholder-informed pre-implementation refinement of a pharmacy-led pre-exposure prophylaxis (PrEP) service delivery model in Malaysia
Source: PLoS One. 2026 Sep 8;21(9):e0357920. doi: 10.1371/journal.pone.0357920 (PMC13552737; doi:10.1371/journal.pone.0357920)
Supplement: Supplementary file 3 [file pone.0357920.s003.pdf]

S3 Appendix. Schedule of activities.

**Schedule of activities for Phase 3 implementation**	**Pre-screening (Pre-consent)**	**Visit 1 Day 1**	**Visit 2 Day 30 ± 7**	**Visit 3 Day 120 ± 7**	**Visit 4 Day 210 ± 7**	**Un-scheduled visit**	**Post-implement-ation**
Identify people at risk of HIV	X						
Refer to researcher for explanation about study using patient information sheet	X						
Assess inclusion/exclusion criteria	X						
Obtain informed consent	X						
Assist client in downloading DOC2US® app and account registration	X						
Begin assessment (initiation) when client arrives at pharmacy		X					
Invite client to complete Section B of assessment (initiation)		X					
Assist client in performing on-site HIV self-testing using oral fluid HIVST kit		X	X	X	X		
Provide PrEP counselling		X					
Determine eligibility for PrEP initiation		X					
Refer to physician if uncertain or ineligible for PrEP initiation or continuation		X	X	X	X	X	
Request electronic prescription for PrEP initiation		X					
Dispense PrEP medication for 30 days		X					
Counsel on dosing, side effects, adherence, risk reduction measures		X	X	X	X	X	
Schedule for follow-up		X	X	X	X	X	
Advise on laboratory testing		X	X	X	X	X	
Begin assessment (follow-up)			X	X	X	X	
Follow-up on laboratory testing & document test results			X	X	X	X	
Assess side effects and adherence			X	X	X	X	
Manage side effects (if mild)			X	X	X	X	
Refer to doctor for serious adverse effects and report to researcher			X	X	X	X	
Refer to doctor if client has consistent low/non-adherence				X	X	X	
Request electronic prescription for PrEP continuation			X	X	X	X	
Dispense PrEP medication for 90 days			X	X	X	X	
Conduct online survey to assess implementation outcomes (clients & pharmacists)							X
Review electronic database to assess adoption/uptake of pharmacy-led PrEP service							X
