# Supplementary material for: Stakeholder-informed pre-implementation refinement of a pharmacy-led pre-exposure prophylaxis (PrEP) service delivery model in Malaysia
Source: PLoS One. 2026 Sep 8;21(9):e0357920. doi: 10.1371/journal.pone.0357920 (PMC13552737; doi:10.1371/journal.pone.0357920)
Supplement: Supplementary file 4 [file pone.0357920.s004.pdf]

S4 Appendix. Assessment tools for pharmacy-led PrEP service initiation and follow-up.

Assessment tool for pharmacy-led PrEP service initiation (Pharmacist searches for the client using their identification card number and clicks "Initiate PrEP" to begin the assessment.)

Section A: Provider details (to be filled by the pharmacist)	
Pharmacy: automated<br>Pharmacist: ______________	Visit date (dd/mm/yy): automated<br>Visit time (start): automated upon clicking the "Initiate PrEP" function

Section B: Client details (to be filled by client)		
1. Personal information:<br>Name : automated if data are available<br>Preferred name : ______________<br>IC: automated if data are available<br>Age : automated if data are available<br>Contact number: automated if data are available<br>Email address : automated if data are available<br>Race/ethnicity :<br>☐ Malay ☐ Chinese ☐ Indian<br>Other : ______________	2. Please indicate your gender:<br>☐ Male<br>☐ Female<br>☐ Trans man<br>☐ Trans woman<br>Other : ______________	3. Kindly indicate your sexual orientation:<br>☐ Heterosexual<br>☐ Gay<br>☐ Lesbian<br>☐ Bisexual<br>Other : ______________
Medical history:	**The following questions will help us understand whether PrEP is right for you.**	
4. Do you have any medical conditions?<br>☐ Yes ☐ No<br>*(If Yes to Q4)*<br>5. Please indicate if you have the following medical condition: *(multiple choice)*<br>☐ Diabetes<br>☐ High blood pressure<br>☐ Kidney disease<br>☐ Other: ______________<br><br>6. Are you currently taking any medications/ supplements?<br>☐ Yes ☐ No<br>*(If Yes to Q6)*<br>7. Please list your current medications/ supplements: ______________<br><br>8. Are you taking bodybuilding supplements containing creatine?<br>☐ Yes ☐ No	18. Please select all that apply to you. *(multiple choice)*<br>☐ I have not heard of PrEP before.<br>☐ I have heard about PrEP but have never taken PrEP before.<br>☐ I have taken PrEP before (more than 6 months ago).<br>☐ I have taken PrEP in the past 6 months.<br>☐ I am currently taking daily PrEP.<br>☐ I am currently taking on-demand PrEP.	21. When did you last have sex? (dd/mm/yy): ______________ **Optional*<br>**Skip if not sexually active.*<br>*If blank, omit Q22-23.*<br><br>22. Was a condom used during your last sex?<br>☐ Yes ☐ No<br><br>23. Total number of sex partners where a condom was not used in the past 3 months:<br>______________

(If select gender: trans woman)
9. Are you taking any estradiol-based hormones?
☐ Yes ☐ No

10. Do you have any drug allergies?
☐ Yes ☐ No

(If Yes to Q10)
11. Please list any drug allergies that you have: ____________

12. When was your last HIV test?
(dd/mm/yy): _____________*Optional*
Skip if no test was done before.
If blank, omit Q13.

13. Result of last HIV test:
☐ Negative ☐ Positive

14. Have you had Hepatitis B infection before?
☐ Yes ☐ No ☐ Not sure
If Yes, refer to physician. If Not sure, advise to go for testing.

15. Have you been vaccinated for Hepatitis B?
☐ Yes ☐ No ☐ Not sure
* *If No / Not sure, advise to go for testing & vaccination.*

(If select gender: female, proceed to Q16 & Q17)
16. Last menstrual period: _______ (dd/mm/yy)
Optional
Skip if no menstrual period in the past 6 months.

17. Please indicate if you are:
☐ Pregnant
☐ Breastfeeding
☐ Using birth control (oral contraceptive pills / implant / vaginal ring / 3-monthly injection / intrauterine device)
☐ None of the above

19. Please select all that apply to you **in the past 6 months.** *(multiple choice)*
☐ History of sexually transmitted infections (syphilis, gonorrhea or chlamydia)
☐ Used stimulant drugs (such as methamphetamine) with condomless sex
☐ Shared injecting materials such as needles, syringes, or other injection equipment
☐ Sold sex to someone
☐ Paid someone for sex
☐ Received post-exposure prophylaxis (PEP)
☐ Sexual partner with HIV
☐ None of the above

(If select sexual partner with HIV in Q19)
20. Is your sexual partner on antiretroviral therapy (ART)?
☐ On ART with undetectable viral load
☐ On ART with detectable viral load
☐ Not on ART
☐ Not sure

24. Did you experience any of the following symptoms **in the past 4 weeks**? *(multiple choice)*
☐ Fever
☐ Sore throat
☐ Swollen glands
☐ Mouth ulcers / oral thrush
☐ Rash
☐ Diarrhea
☐ None of the above
* *Fever plus 2 or more other symptoms in the past 4 weeks with reported high-risk exposure warrants referral to a physician.*

25. Are you **currently** experiencing any of the following symptoms?
(multiple choice)
☐ Rashes / ulcers / blisters in genital area
☐ Discharge from vagina or penis
☐ None of the above

(display summary of Section B for pharmacists to view)
* *Probe further on medical or sexual history if needed.*

Section C: Screening for HIV & pregnancy (Section C – F) to be filled by pharmacist)

HIV self-test result: ☐ Non-reactive ☐ Reactive
Lot number of test kit: _______ Expiry date of test kit: _______ (dd/mm/yy)

(If select gender: female) * *If sexually active and last menstrual period is late by 4 weeks in the absence of contraception / condom, suggest urine pregnancy test.*
Was a pregnancy test performed? ☐ Yes ☐ No
(If Yes) Pregnancy test result: ☐ Negative ☐ Positive

Section D: Referral to physician

Do not initiate PrEP and refer to physician if any of the following applies: *(multiple choice)*
☐ Symptoms of HIV seroconversion ☐ Reactive HIV test result ☐ Exposure to HIV (within 72 hours)* ☐ Hepatitis B infection
☐ Kidney-related comorbidities/risk factors ☐ Pregnant ☐ Breastfeeding ☐ Allergic to TDF/FTC
* *Exposure to HIV-positive partner not on treatment or on treatment with detectable viral load (within 72 hours) - to assess need for PEP.*
☐ None of the above (proceed to initiation)
☐ Acute STI symptoms (proceed to initiation and provide referral to physician)

[If any of the above options is selected, this message will appear: "Assessment was terminated due to a detected red flag. Please refer to a physician immediately."]

Section E: PrEP counselling

The following are counselling points that should be covered (check if you have counselled):
☐ Indication ☐ Effectiveness ☐ Safety ☐ Side effects ☐ HIV testing ☐ Dosing ☐ Adherence
☐ Interactions ☐ Guidance on starting/stopping PrEP ☐ Risk reduction measures

**Section F: PrEP initiation**
Indication for PrEP:<br>☐ At risk of HIV<br>☐ Request for PrEP although no risk of HIV was reported<br>☐ Request for PrEP due to anticipated risk in the future (condomless sex or sharing injecting materials)<br><br>Willing to use PrEP:<br>☐ Yes ☐ No [If No, assess reasons and offer to schedule an appointment for future consultation.]<br>Reason for not willing to use PrEP (please describe): ______________ **optional*<br>*[If No is selected, this message will appear: "Assessment was terminated due to unwillingness to use PrEP. Please offer to schedule appointment for future consultation."]*
PrEP dosing:<br>☐ Daily ☐ On-demand (only for cis-gender men & trans women not taking estradiol-based hormones, with sexual exposure)
Next pharmacy appointment date: ______________(dd/mm/yy)
Recommended baseline lab tests (provide information on nearest lab/clinic & advise to bring lab results during next visit) *(multiple choice)*<br>☐ Kidney function test ☐ Liver function test ☐ STI screening ☐ Hepatitis B ☐ Hepatitis C
Request physician for electronic prescription:<br>☐ Yes (30-day supply of TDF/TFC for new or restarting PrEP user)<br>☐ Yes (90-day supply of TDF/TFC for continuing PrEP user – only if they reported current use and last HIV-negative test result was within 3 months)<br>*[Back Function to edit previous information]*
Visit time (end): automated when "Mark as supplied" is clicked

Assessment tool for follow-up (Pharmacist searches for the client using their identification card number and clicks "Refill PrEP" to begin the assessment.)

Section A: Provider details (Section A-I to be filled by pharmacist)

Pharmacy: automated<br>Pharmacist:	Visit date (dd/mm/yy): automated<br>Visit time (start): automated upon clicking "Refill PrEP" function

Section B: Client details

Name: automated IC: automated PrEP dosing: ☐ Daily ☐ On-demand

Section C: PrEP side effects, adverse events, kidney function & hepatitis B

Side effects: ☐ No ☐ Yes (please list): ______________
Date of side effects started: __________ (dd/mm/yy) *optional Duration of side effects: ________ (days) *optional
Management of side effects (please describe): ______________*optional

Serious adverse events: ☐ No ☐ Yes (please list): ______________

Did the client get a kidney function test after the last visit? ☐ Yes ☐ No
(If Yes) Date of last kidney function test: __________ (dd/mm/yy) eGFR value: ____________ mL/min per $1.73m^2$

Did the client get a hepatitis B test after the last visit? ☐ Yes ☐ No
(If Yes) Date of last hepatitis B test: _______ (dd/mm/yy)
HBsAg: ☐ Detected ☐ Not detected HBsAb: _____ IU/L *optional **If HBsAb <10, refer to physician for booster dose.*

Section D: HIV seroconversion & STI symptoms

Did the client experience any of the following symptoms **in the past 4 weeks**? *(multiple choice)*
☐ Fever ☐ Sore throat ☐ Swollen glands ☐ Mouth ulcers / oral thrush ☐ Rash ☐ Diarrhea
☐ None of the above
** Fever + 2 or more other symptoms in the past 4 weeks with reported high-risk exposure warrants referral to a physician.*

Is the client **currently** experiencing any of the following symptoms? *(multiple choice)*
☐ Rashes / ulcers / blisters in genital area ☐ Discharge from vagina or penis ☐ None of the above

Section E: Screening for HIV & Pregnancy

HIV self-test result: ☐ Non-reactive ☐ Reactive
Lot number of test kit: _______ Expiry date of test kit: _______ (dd/mm/yy)

(If select gender: female) ** If sexually active and last menstrual period is late by 4 weeks in the absence of contraception/condom, suggest urine pregnancy test.*
Was a pregnancy test performed? ☐ Yes ☐ No
(If Yes) Pregnancy test result: ☐ Negative ☐ Positive

Section F: Change in HIV risk

Did the client report change in HIV risk behaviours compared to what was reported in previous visit?
☐ No ☐ Yes (please describe): ______________

Section G: PrEP adherence

Is the client still taking PrEP?
☐ Yes ☐ No (assess reasons of stopping PrEP)

(If Yes)
** Assess how did he/she take PrEP in the past month, how he/she remember to take PrEP, and frequency of missed dose.*
In the past 4 weeks, what percent of the time was the client able to take all his/her PrEP medication?
______________ (0% - 100%)

* If intermediate (40-80%) or low (<40%) PrEP adherence, assess reasons for non-adherence and provide adherence counselling.
Reasons for non-adherence (please describe): ______________
Adherence counselling provided: ☐ Yes ☐ No

(If No)
Reasons of stopping PrEP (please describe): ______________

Section H: Referral to physician

Do not continue PrEP and refer to physician if any of the following applies: *(multiple choice)*
☐ Serious adverse events ☐ Symptoms of HIV seroconversion ☐ Reactive HIV test result ☐ Pregnant
☐ eGFR <60 mL/min per $1.73m^2$ ☐ No baseline kidney function test (within 3 months after initiation) ☐ Hepatitis B infection
☐ Need for PEP ☐ Daily PrEP: <4 tablets/week (men) / <6 tablets/week (women) ☐ On-demand PrEP: any missed dose
☐ None of the above (proceed to continuation)
☐ Acute STI symptoms (proceed to continuation and provide referral to physician)

[If any of the above options is selected, this message will appear: "Assessment was terminated due to a detected red flag. Please refer to a physician immediately."]

**Section I: PrEP continuation**
Willing to continue PrEP: ☐ Yes ☐ No (assess reasons of stopping PrEP)<br>Reasons for stopping PrEP (please describe): _____________<br>*[If No is selected, this message will appear: "Assessment was terminated due to unwillingness to use PrEP. Please offer to schedule appointment for future consultation."]*
PrEP dosing:<br>☐ Daily ☐ On-demand (only for cis-gender men & trans women not taking estradiol-based hormones, with sexual exposure)
Next pharmacy appointment date: _____________(dd/mm/yy)
Recommended lab tests (provide information on nearest lab/clinic & advise to bring lab results during next visit) *(multiple choice)*<br>☐ Kidney function test ☐ STI screening ☐ Hepatitis B ☐ Hepatitis C ☐ None of the above
Request physician for electronic prescription (90-day supply of TDF/TFC): ☐ Yes *[Back Function to edit previous information]*
Visit time (end): automated when "Mark as supplied" is clicked
