# Supplementary material for: Stakeholder-informed pre-implementation refinement of a pharmacy-led pre-exposure prophylaxis (PrEP) service delivery model in Malaysia
Source: PLoS One. 2026 Sep 8;21(9):e0357920. doi: 10.1371/journal.pone.0357920 (PMC13552737; doi:10.1371/journal.pone.0357920)
Supplement: Supplementary file 5 [file pone.0357920.s005.pdf]

S5 Appendix: Stakeholder feedback on pilot study procedures, care pathway, workflow, schedule of activities, and assessment tools.

**Topics**	**Stakeholder feedback**
**Pilot study procedures**	**Inclusion and exclusion criteria:**<br>• Allow pharmacist-assisted consent for clients who do not understand English or Malay.<br>• Refer individuals who do not meet study criteria to nearby PrEP providers to ensure continued access to HIV prevention services.<br>**Eligibility for PrEP initiation and continuation:**<br>• Clients using bodybuilding supplements containing creatine may initiate PrEP but should be counselled on kidney function monitoring needs based on baseline results and individual risk factors.<br>**Follow-up timepoints for new, restarting and continuing PrEP users:**<br>• New users: first follow-up 1 month after baseline visit to account for the HIV self-test window period, assess side effects, and support adherence, then every 3 months thereafter.<br>• Restarting users: individuals who have not used PrEP in the past 6 months; follow the same schedule as above (1 month, then every 3 months).<br>• Continuing users: individuals who have used PrEP within the past 6 months; follow the same schedule as above (1 month, then every 3 months).<br>• Continuing users who self-report a negative HIV test within the past 3 months may be scheduled for 3 monthly follow up without the month-1 follow-up visit after the baseline assessment.<br>**Referral to physician:**<br>• Pharmacists should first assess whether reported “allergies” are true allergic reactions or expected side effects before making a referral.<br>• Refer clients for post-exposure prophylaxis assessment if they report possible HIV exposure with missed PrEP doses (for daily PrEP, fewer than 4 tablets in the past 7 days or missing more than 7 consecutive days; for on demand PrEP, any missed dose during the dosing cycle).<br>**Management of reactive HIV self-test (HIVST) results:**<br>• Reassure clients that HIV self-testing at the pharmacy is a screening test and not diagnostic.<br>• All reactive HIVST results must be followed by confirmatory testing with a trained medical provider.<br>• Advise clients with reactive HIVST results to seek confirmatory testing at a community-friendly PrEP clinic or obtain assistance from community health workers for free testing and psychosocial support at public healthcare facilities.<br>• Pharmacists should notify the study team of any reactive HIVST result.<br>• The study team should follow up with the client within one week to ensure confirmatory testing was completed and that the client is linked to appropriate care.
**Care pathway**	Accepted.

**Workflow**	Accepted.
**Schedule of activities**	Accepted.
**Assessment tools**	**Personal information:**<br><br>• Include a field for preferred name to allow clients to use their chosen name instead of their registered name.<br>**Symptoms of HIV seroconversion:**<br><br>• Refer to a physician if a client reports fever plus 3 or more symptoms and high-risk exposure within the past 4 weeks.<br>• Relevant symptoms include sore throat, swollen glands, mouth ulcers or oral thrush, rash, and diarrhea.<br>• Exclude body aches, as they are non-specific and may lead to unnecessary referrals.<br>• Pharmacists should ask whether the client has seen a physician in the past 4 weeks before initiating a referral.<br>**Sexually transmitted infections (STI) screening by symptoms:**<br><br>• Conduct STI symptom screening at every visit and include it in the assessment tool.<br>• Any reported symptoms should prompt further probing and referral if needed.<br>**Adherence:**<br><br>• Begin with a subjective adherence assessment (for example, how PrEP was taken in the past month, reminders used, and frequency of missed doses).<br>• Follow with a percentage-based assessment (“In the past 4 weeks, what percentage of the time were you able to take all your PrEP doses?”) and record this in the assessment tool.<br>• Provide adherence counselling for clients with low adherence for the first time.<br>• Refer to a physician if low adherence persists (for example, twice consecutively) and the client reports high risk exposure since the last visit.
