# Supplementary material for: Stakeholder-informed pre-implementation refinement of a pharmacy-led pre-exposure prophylaxis (PrEP) service delivery model in Malaysia
Source: PLoS One. 2026 Sep 8;21(9):e0357920. doi: 10.1371/journal.pone.0357920 (PMC13552737; doi:10.1371/journal.pone.0357920)
Supplement: Supplementary file 6 [file pone.0357920.s006.pdf]

S6 Appendix. Malaysia-specific implementation considerations and adaptations for pharmacy-led PrEP service delivery.

Implementation domain	Malaysia-specific regulatory, policy, and health system context	Implications for pharmacy-led PrEP service delivery	Adaptations used in this study
**PrEP prescribing authority**	Oral TDF/FTC classified as Group B poison under the Poisons Act 1952; requires a prescription by a registered medical practitioner	Pharmacists cannot independently prescribe PrEP	Telemedicine-enabled physician prescribing integrated into pharmacy workflow to enable same-day access
**HIV testing in private pharmacies**	Rapid HIV testing not permitted in private pharmacies; only assisted HIV self-testing (HIVST) allowed	Limited ability to perform point-of-care diagnostic testing	Provider-assisted HIVST used for screening, with clear referral pathways for confirmatory testing
**HIV case notification**	Only confirmed HIV diagnoses by physicians are notifiable to MOH	Pharmacists unclear about reporting responsibilities	Reactive HIVST managed as screening only; referral to physicians for confirmatory testing and notification
**Laboratory services**	Community pharmacies lack on-site laboratory testing facilities	Difficulty conducting baseline and follow-up tests	Partnerships established with nearby clinics and laboratories for kidney function testing, hepatitis screening, and sexually transmitted infections (STI) screening.
**Pharmacist scope of practice**	Pharmacists authorized to dispense and counsel but not diagnose or prescribe	Requires task delineation and safeguards	Use of standardized assessment tools, referral triggers, and physician oversight
**Data protection & confidentiality**	Personal Data Protection Act (PDPA) applies to private healthcare data	High sensitivity of HIV-related information	Secure telemedicine platform with restricted access to client data and role-based permissions
**Advertising & promotion of services**	Restrictions on pharmacy advertising of medical services	Limits broad public promotion of PrEP services	Targeted promotion via NGOs, social media, and community-based outreach rather than mass advertising
**Training and accreditation**	No national accredited PrEP training for pharmacists	Variability in pharmacist preparedness	Study-specific standardized online training program and an in-person workshop implemented.
**Financing & reimbursement**	Private pharmacy services are provided on an out-of-pocket basis; no MOH reimbursement is available.	Affordability and sustainability concerns	Temporary medication rebates during the pilot; advocacy for public–private reimbursement mechanisms
