# Supplementary material for: Stakeholder-informed pre-implementation refinement of a pharmacy-led pre-exposure prophylaxis (PrEP) service delivery model in Malaysia
Source: PLoS One. 2026 Sep 8;21(9):e0357920. doi: 10.1371/journal.pone.0357920 (PMC13552737; doi:10.1371/journal.pone.0357920)
Supplement: Supplementary file 7 [file pone.0357920.s008.docx]

**Professor Frederick L. Altice**

Professor of Medicine, Epidemiology, and Public Health

Yale School of Medicine

Yale University, United States of America

frederick.altice@yale.edu

25^th^ June 2026

**Editor-in-Chief
PLOS ONE**

**Subject: Response to Reviewers for Manuscript Revision (PONE-D-26-19386)**

Dear Dr. Ali Haider Mohammed,

We sincerely appreciate the opportunity to revise our manuscript, titled "Stakeholder-informed pre-implementation design of a pharmacy-led pre-exposure prophylaxis (PrEP) service delivery model in Malaysia" (Manuscript ID: PONE-D-26-19386).

We are grateful for the constructive feedback provided by the academic editor and reviewers. We have carefully addressed each comment and have made the necessary revisions to enhance the clarity and quality of our manuscript. Below, we provide a point-by-point response to each comment, detailing the changes made.

# Journal requirements:

**Comment 1:** Please ensure that your manuscript meets PLOS ONE's style requirements, including those for file naming. The PLOS ONE style templates can be found at <https://journals.plos.org/plosone/s/file?id=wjVg/PLOSOne_formatting_sample_main_body.pdf> and [https://journals.plos.org/plosone/s/file?id=ba62/PLOSOne_formatting_sample_title_authors_affiliations.pdf](https://track.editorialmanager.com/CL0/https:%2F%2Fjournals.plos.org%2Fplosone%2Fs%2Ffile%3Fid=ba62%2FPLOSOne_formatting_sample_title_authors_affiliations.pdf/1/010f019e5d29b399-4b86f61a-2b50-4bc3-bfc9-ce9afbe774e8-000000/jkFWRp5xMUI9vUfvILu7wccLnrTzZTbJqQ0aqSZQf7E=258)

**Response:** Thank you for highlighting the journal formatting requirements. We have carefully revised the manuscript to ensure compliance with PLOS ONE style requirements, including formatting, author affiliations, corresponding author formatting, supporting information labeling, and file naming conventions, in accordance with the journal guidelines and templates.

**Comment 2:** Thank you for stating in your Funding Statement:

[This research was funded by the World Health Organization (WHO) (WHO registration: 2023/1363112-0) – Principal Investigators (PI): Iskandar Azwa <https://www.who.int/malaysia/> and the Malaysian Implementation Science Training program (MIST) for YG supported by Fogarty International Center, NIDA, NIMH and NICHD (D43 TW-011324 – PI: Frederick L. Altice, Adeeba Kamarulzaman) <https://www.fic.nih.gov/.The> funders had no role in study design, information collection and analysis, decision to publish, or preparation of the manuscript.].

Please provide an amended statement that declares *all* the funding or sources of support (whether external or internal to your organization) received during this study, as detailed online in our guide for authors at <http://journals.plos.org/plosone/s/submit-now>.  Please also include the statement “There was no additional external funding received for this study.” in your updated Funding Statement.

Please include your amended Funding Statement within your cover letter. We will change the online submission form on your behalf.

**Response:** We have revised the Funding Statement to include all sources of funding and support received for this study. The revised Funding Statement is as follows:

“This activity was funded by the World Health Organization (WHO) (WHO registration: 2023/1363112-0; Principal Investigator: Iskandar Azwa) https://www.who.int/malaysia/ and the Malaysian Implementation Science Training program (MIST) for YG, supported by the Fogarty International Center, NIDA, NIMH, and NICHD (D43 TW-011324; Principal Investigators: Frederick L. Altice and Adeeba Kamarulzaman) https://www.fic.nih.gov/. The funders had no role in study design, information collection and analysis, decision to publish, or preparation of the manuscript. There was no additional external funding received for this study. The Red Clinic provided salary support to author AY in the form of employment but did not provide funding for this study and had no additional role in study design, information collection and analysis, decision to publish, or preparation of the manuscript. The specific role of this author is articulated in the ‘author contributions’ section.”

We have included the amended Funding Statement in our cover letter.

**Comment 3:** Thank you for stating the following in the Competing Interests section:

[The authors have declared that no competing interests exist.].

We note that one or more of the authors are employed by a commercial company: The Red Clinic.

1. Please provide an amended Funding Statement declaring this commercial affiliation, as well as a statement regarding the Role of Funders in your study. If the funding organization did not play a role in the study design, data collection and analysis, decision to publish, or preparation of the manuscript and only provided financial support in the form of authors' salaries and/or research materials, please review your statements relating to the author contributions, and ensure you have specifically and accurately indicated the role(s) that these authors had in your study. You can update author roles in the Author Contributions section of the online submission form.

Please also include the following statement within your amended Funding Statement.

“The funder provided support in the form of salaries for authors [insert relevant initials], but did not have any additional role in the study design, data collection and analysis, decision to publish, or preparation of the manuscript. The specific roles of these authors are articulated in the ‘author contributions’ section.”

If your commercial affiliation did play a role in your study, please state and explain this role within your updated Funding Statement.

2. Please also provide an updated Competing Interests Statement declaring this commercial affiliation along with any other relevant declarations relating to employment, consultancy, patents, products in development, or marketed products, etc.

Within your Competing Interests Statement, please confirm that this commercial affiliation does not alter your adherence to all PLOS ONE policies on sharing data and materials by including the following statement: "This does not alter our adherence to PLOS ONE policies on sharing data and materials.” (as detailed online in our guide for authors <http://journals.plos.org/plosone/s/competing-interests>) . If this adherence statement is not accurate and there are restrictions on sharing of data and/or materials, please state these. Please note that we cannot proceed with consideration of your article until this information has been declared.

Please include both an updated Funding Statement and Competing Interests Statement in your cover letter. We will change the online submission form on your behalf.

**Response:** We have updated both the Funding Statement and Competing Interests Statement in our cover letter to clarify the commercial affiliation identified by the journal.

The revised Competing Interests Statement is as follows:

“AY is affiliated with The Red Clinic, a private healthcare provider in Malaysia, and participated in this study in his professional capacity as a private-sector PrEP prescriber and committee member of the professional society Malaysian Society for HIV Medicine (MASHM). The Red Clinic provided salary support to author AY in the form of employment but did not provide funding for this study and had no additional role in study design, information collection and analysis, decision to publish, or preparation of the manuscript. The remaining authors declare that no competing interests exist. This does not alter our adherence to PLOS ONE policies on sharing data and materials.”

**Comment 4:** Please include captions for your Supporting Information files at the end of your manuscript, and update any in-text citations to match accordingly. Please see our Supporting Information guidelines for more information: [http://journals.plos.org/plosone/s/supporting-information](http://track.editorialmanager.com/CL0/http:%2F%2Fjournals.plos.org%2Fplosone%2Fs%2Fsupporting-information/1/010f019e5d29b399-4b86f61a-2b50-4bc3-bfc9-ce9afbe774e8-000000/uMYFseTlA_XJK4va4t7pNf0ATQ42G8KE17fUqXU59FM=258).

**Response:** We have reviewed the Supporting Information files, captions, file names, and corresponding in-text citations to ensure consistency with PLOS ONE Supporting Information guidelines. Supporting Information captions are provided at the end of the manuscript, and all Supporting Information files and citations have been updated accordingly.

**Comment 5:** We note that Figures S6.1 and S6.2 in your submission contain copyrighted images. All PLOS content is published under the Creative Commons Attribution License (CC BY 4.0), which means that the manuscript, images, and Supporting Information files will be freely available online, and any third party is permitted to access, download, copy, distribute, and use these materials in any way, even commercially, with proper attribution. For more information, see our copyright guidelines: <http://journals.plos.org/plosone/s/licenses-and-copyright>.

We require you to either (1) present written permission from the copyright holder to publish these figures specifically under the CC BY 4.0 license, or (2) remove the figures from your submission:

1. You may seek permission from the original copyright holder of Figures S6.1 and S6.2 to publish the content specifically under the CC BY 4.0 license.

We recommend that you contact the original copyright holder with the Content Permission Form (<http://journals.plos.org/plosone/s/file?id=7c09/content-permission-form.pdf>) and the following text:

“I request permission for the open-access journal PLOS ONE to publish XXX under the Creative Commons Attribution License (CCAL) CC BY 4.0 (<http://creativecommons.org/licenses/by/4.0/>). Please be aware that this license allows unrestricted use and distribution, even commercially, by third parties. Please reply and provide explicit written permission to publish XXX under a CC BY license and complete the attached form.”

Please upload the completed Content Permission Form or other proof of granted permissions as an "Other" file with your submission.

In the figure caption of the copyrighted figure, please include the following text: “Reprinted from [ref] under a CC BY license, with permission from [name of publisher], original copyright [original copyright year].”

2. If you are unable to obtain permission from the original copyright holder to publish these figures under the CC BY 4.0 license or if the copyright holder’s requirements are incompatible with the CC BY 4.0 license, please either i) remove the figure or ii) supply a replacement figure that complies with the CC BY 4.0 license. Please check copyright information on all replacement figures and update the figure caption with source information. If applicable, please specify in the figure caption text when a figure is similar but not identical to the original image and is therefore for illustrative purposes only.

If the reviewer comments include a recommendation to cite specific previously published works, please review and evaluate these publications to determine whether they are relevant and should be cited. There is no requirement to cite these works unless the editor has indicated otherwise.

**Response:** Thank you for highlighting the copyright requirements for the Supporting Information figures. Figures S6.1 and S6.2 have been removed from the Supporting Information to comply with PLOS ONE copyright and licensing policies. We have also revised the Methods section to clarify that the promotional and educational materials were reviewed and refined during the stakeholder consultation but are not included as Supporting Information.

**Comment 6:** The manuscript repeatedly states the consultation was “not qualitative research” but simultaneously uses discussions, field notes, structured worksheets, descriptive synthesis, and “member checking.” This creates conceptual confusion.

**Response:** We acknowledge that several approaches used in the stakeholder consultation, including facilitated discussions, field notes, descriptive synthesis, and stakeholder validation, share similarities with qualitative engagement approaches and may have created conceptual ambiguity in the original manuscript. To improve clarity, we have revised the manuscript to more clearly describe the activity as an implementation-informed stakeholder engagement activity conducted to support pre-implementation planning and refinement of the pharmacy-led PrEP service delivery model.

While the consultation incorporated discussion-based stakeholder engagement approaches, it was not designed or conducted as a formal qualitative research study intended to generate theory-driven or generalizable qualitative findings. We also revised the manuscript to clarify the purpose and synthesis of stakeholder inputs and replaced the term “member checking” with “feedback, accuracy checking, and validation” to better reflect the stakeholder review process used in this consultation. Relevant revisions were made throughout the Materials and methods, including ethical approval section, to improve conceptual clarity and methodological transparency.

**Comment 7:** Limited analytical depth. The “Results” section is largely descriptive listing of barriers and solutions. There is: no prioritization framework, no ranking of implementation barriers, no systematic coding structure, no explicit implementation determinant mapping (e.g., CFIR domains), limited synthesis beyond narrative summaries. For implementation science audiences, this weakens rigor. The discussion repeatedly states phrases like: “This reflects an implementation determinant…” This becomes formulaic and reads somewhat AI-assisted/editorially repetitive. The manuscript could likely be reduced by 20–25% without losing substance.

**Response:** We acknowledge that the stakeholder consultation was designed as a pragmatic, implementation-informed consultation to support pre-implementation planning and refinement of the pharmacy-led PrEP service delivery model, rather than as a formal determinant-mapping or theory-generating implementation science study. Accordingly, the Results section focused on summarizing stakeholder-proposed solutions and synthesizing key implementation considerations relevant to real-world service planning.

To address concerns regarding analytical depth, we clarified in the Methods, Results, and Discussion that the consultation built on findings from a preceding exploratory phase that identified barriers and facilitators to pharmacy-led PrEP implementation, whereas the consultation itself was intended to inform implementation planning and refine the service model for pilot implementation. We also removed terminology such as “implementation determinant” that could imply formal determinant identification or mapping and revised the Discussion to focus on stakeholder-informed implementation considerations rather than implementation framework interpretation.

To strengthen synthesis, we clarified how the key implementation considerations were derived from recurring issues identified across multiple consultation activities, including discussions on proposed solutions, workflow design, pilot procedures, assessment tools, training requirements, and site preparation. We also revised Table 1 (title and legend) to better reflect the relationship between barriers identified during the preceding exploratory phase and stakeholder-proposed solutions, and to explicitly note that the consultation did not include formal prioritization or ranking of barriers or solutions. We also expanded the limitations section to explicitly acknowledge the absence of formal prioritization methods and qualitative coding procedures, and to discuss their implications for interpretation of the findings.

Finally, we improved clarity and readability throughout the Results and Discussion by reducing repetitive implementation science phrasing, streamlining narrative summaries, strengthening integration of stakeholder inputs across implementation considerations, and condensing several descriptive sections. These revisions were undertaken to better align the methodological approach, analysis, and interpretation with the pragmatic and formative purpose of the consultation.

**Comment 8:** Potential selection bias. Stakeholders were heavily drawn from implementation partners already committed to the pilot: Alpro Pharmacy, DOC2US,
collaborating clinicians/NGOs. This risks: confirmation bias, overly favorable feasibility perceptions, underrepresentation of dissenting regulatory or conservative pharmacy perspectives. The limitation section acknowledges this, but only briefly.
I would want deeper critical reflection. The manuscript sometimes overextends implications for “Malaysia” broadly despite likely limited rural applicability.

**Response:** Thank you for this comment. We agree that the stakeholder group was intentionally composed of individuals and organizations involved in, or supportive of, implementation of the pilot study, which may have introduced selection and confirmation bias. To address this concern, we expanded the limitations section to provide a more critical discussion of the potential underrepresentation of stakeholders with differing regulatory, professional, or implementation perspectives, and the implications for interpretation of the findings.

We also clarified the contextual boundaries of the consultation and pilot study. The consultation was conducted to inform implementation of an urban, private-sector pharmacy-led PrEP model in the Klang Valley and was not intended to represent all pharmacy settings in Malaysia. Accordingly, we revised the manuscript to avoid overgeneralization and acknowledge that implementation challenges, resource availability, and service delivery considerations may differ in rural settings, regions with more limited healthcare infrastructure, and pharmacy settings without established telemedicine and physician-prescribing partnerships. These clarifications have been incorporated into the limitations and Conclusion sections.

**Comment 9:** Some implementation claims are speculative. Statements about: scalability, affordability, sustainability, public-private reimbursement, workflow feasibility are not empirically tested here. They are stakeholder perceptions. The authors should more explicitly distinguish: stakeholder opinion, vs demonstrated implementation outcomes.

**Response:** Thank you for highlighting this. We agree that the stakeholder consultation was designed to elicit stakeholder perspectives, recommendations, and implementation considerations rather than to evaluate implementation outcomes. As such, issues such as affordability, sustainability, scalability, reimbursement mechanisms, and workflow feasibility should be interpreted as stakeholder-informed considerations for implementation planning rather than empirically demonstrated outcomes.

To address this concern, we reviewed the Results, Discussion, and Conclusion sections and revised wording where necessary to more clearly distinguish stakeholder perspectives from observed or measured outcomes. In particular, we replaced definitive statements regarding feasibility, scalability, sustainability, and workflow feasibility with language reflecting stakeholder recommendations, perceived opportunities, or anticipated implementation considerations. We also revised the manuscript to distinguish stakeholder perceptions and recommendations from implementation outcomes, which remain to be evaluated during pilot implementation.

**Reviewer’s comments:**

**Reviewer's Responses to Questions**

**Comments to the Author**

1. Is the manuscript technically sound, and do the data support the conclusions?

The manuscript must describe a technically sound piece of scientific research with data that supports the conclusions. Experiments must have been conducted rigorously, with appropriate controls, replication, and sample sizes. The conclusions must be drawn appropriately based on the data presented.

Reviewer #1: Partly

Reviewer #2: Yes

2. Has the statistical analysis been performed appropriately and rigorously?

Reviewer #1: N/A

Reviewer #2: N/A

3. Have the authors made all data underlying the findings in their manuscript fully available?

The [PLOS Data policy](http://track.editorialmanager.com/CL0/http:%2F%2Fwww.plosone.org%2Fstatic%2Fpolicies.action%23sharing/1/010f019e5d29b399-4b86f61a-2b50-4bc3-bfc9-ce9afbe774e8-000000/vHb7MzV_owe5VB9AQ1JSA9VbsKQmUnxe3eH9vP9ayZM=258) requires authors to make all data underlying the findings described in their manuscript fully available without restriction, with rare exception (please refer to the Data Availability Statement in the manuscript PDF file). The data should be provided as part of the manuscript or its supporting information, or deposited to a public repository. For example, in addition to summary statistics, the data points behind means, medians and variance measures should be available. If there are restrictions on publicly sharing data—e.g. participant privacy or use of data from a third party—those must be specified.

Reviewer #1: Yes

Reviewer #2: Yes

4. Is the manuscript presented in an intelligible fashion and written in standard English?

PLOS ONE does not copyedit accepted manuscripts, so the language in submitted articles must be clear, correct, and unambiguous. Any typographical or grammatical errors should be corrected at revision, so please note any specific errors here.

Reviewer #1: Yes

Reviewer #2: Yes

**5. Review Comments to the Author**

# Response to Reviewer #1:

**Comment 1:** The proposed service targets key populations such as MSM, transgender women, and people who inject drugs, yet none of the consultation participants directly represented these groups. Although the authors referred to prior formative interviews, the consultation itself was largely provider- and system-focused. Therefore, conclusions related to privacy, stigma, acceptability, and service preferences should be interpreted cautiously. This limitation should be stated more explicitly in the manuscript.

**Response:** Thank you for this comment. We acknowledge that the role of community-based organizations and their representatives was not described sufficiently clearly in the original manuscript. To address this, we have revised the Methods section to clarify that the stakeholder consultation included representatives from several community-based organizations serving key populations affected by HIV in Malaysia, including the Malaysian AIDS Council, PT Foundation, Kuala Lumpur AIDS Support Services Society (KLASS), SEED Foundation, and Persatuan Insaf Murni Malaysia (PIMM). These organizations work closely with key populations, including men who have sex with men, transgender women, and people who inject drugs, and contributed community perspectives during the consultation. The representatives were also community health workers from key population communities, with lived and/or professional experience supporting HIV prevention and care services within these populations.

In addition, the consultation built on findings from the preceding exploratory phase, which involved in-depth interviews with key populations, community pharmacists, and PrEP prescribers. Findings from these interviews, including barriers and facilitators related to privacy, stigma, service preferences, and PrEP access, were presented during the consultation and informed stakeholder discussions and solution development. However, we acknowledge that the consultation did not include direct participation from a broader group of individual end-users and that perspectives on privacy, stigma, acceptability, and service preferences should therefore be interpreted alongside findings from the exploratory phase. To address this concern, we have clarified the role of community-based organizations in the Methods section and strengthened the limitations section accordingly.

**Comment 2:** Several major barriers identified in the manuscript, including prescribing restrictions, Group B Poison classification of TDF/FTC, HIV testing regulations, and reimbursement policies, fall directly under MOH authority. Many proposed long-term solutions in Table 1 would require involvement from the Disease Control Division and Pharmacy Programme. In addition, there appears to be inconsistency between the methods section, which states MOH representatives were not invited, and the acknowledgements section listing MOH specialists. This should be clarified.

**Response:** Thank you for this comment. We agree that several barriers identified during the consultation, including prescribing restrictions, HIV testing regulations, medication classification, and financing mechanisms, involve policy and regulatory issues that fall within the remit of the Ministry of Health (MOH). However, the primary purpose of the consultation was to inform implementation planning and refinement of a pharmacy-led PrEP service model for pilot implementation within participating private-sector sites operating under existing regulatory frameworks. As such, stakeholder selection prioritized individuals directly involved in pilot implementation, service delivery, clinical care, and community engagement. While long-term solutions identified by stakeholders may require broader policy and regulatory changes, the consultation focused primarily on developing practical approaches that could be implemented within the existing regulatory environment. We have clarified this rationale in the Methods section and acknowledged in the limitations section that participation from the MOH Disease Control Division and Pharmacy Programme could have provided additional perspectives on regulatory alignment and national scale-up pathways.

We also acknowledge that the original manuscript may have created confusion regarding MOH involvement. Representatives from the MOH Disease Control Division and MOH Pharmacy Programme were not invited in their official programmatic or regulatory capacities. However, two MOH specialists attended the consultation as PrEP prescribers and clinical experts and were therefore included within the prescriber stakeholder group, which comprised prescribers from both the public and private sectors, rather than as MOH representatives. We have revised the Methods section to clarify this distinction.

**Comment 3:** Only one pharmacy chain and one telemedicine provider participated in the consultation. As a result, the proposed workflow and operational recommendations may reflect the systems and resources already available within these organisations. Independent pharmacies and smaller chains may face different challenges. The authors should avoid overgeneralising the findings to all Malaysian community pharmacies.

**Response:** Thank you for this comment. We agree that the proposed workflow and operational recommendations were informed by stakeholders involved in the participating implementation partners, namely Alpro Pharmacy and DOC2US^®^, and may therefore reflect the implementation context of a pharmacy-led PrEP service model supported by established telemedicine and physician-prescribing partnerships.

To address this concern, we strengthened the limitations section to clarify that the consultation was conducted to support implementation of an urban, private-sector pharmacy-led PrEP service model in the Klang Valley and that the findings may not fully reflect implementation considerations in other settings, including pharmacy settings without established telemedicine and physician-prescribing partnerships. In addition, we clarified the implementation context in the Methods section by describing the roles of Alpro Pharmacy and DOC2US^®^ as the participating implementation partners. The Discussion and Conclusion sections were also revised to avoid overgeneralization and emphasize that the findings are intended to inform pilot implementation and future adaptation of pharmacy-led PrEP services in similar settings rather than represent all community pharmacies in Malaysia.

**Comment 4:** The manuscript states that stakeholder inputs were synthesised descriptively without formal qualitative analysis. However, the paper still presents thematic and interpretive conclusions, which creates some methodological inconsistency. Additional details are needed, including:

- facilitator roles and positioning
- whether discussions were audio-recorded or only field-noted
- how convergence of ideas was determined
- how disagreements were managed
- the process and outcomes of member checking

The authors may also consider using a reporting framework such as GRIPP2-SF, SRQR, or COREQ to improve transparency.

**Response:** We thank the reviewer for suggesting GRIPP2-SF, SRQR, and COREQ. We reviewed these reporting frameworks. However, as the consultation was conducted primarily as an implementation-informed stakeholder engagement activity rather than a formal qualitative study, we did not adopt a specific qualitative or patient and public involvement reporting framework. Instead, we have incorporated additional methodological details to improve transparency regarding facilitator roles, documentation procedures, synthesis of stakeholder inputs, management of differing views, and stakeholder validation.

**Comment 5:** Table 1 contains many suggested solutions, but no formal prioritisation method was applied. Therefore, the findings only show that stakeholders identified these issues, not which were considered most important or most feasible. The process used to derive the ten “key implementation considerations” in Figure 3 should also be described more clearly.

**Response:** Thank you for this comment. We agree that the consultation did not include a formal prioritization process and therefore cannot determine the relative importance of identified barriers, solutions, or implementation strategies. Rather, the consultation was intended to support implementation planning and service refinement by identifying stakeholder-proposed short- and long-term solutions relevant to pilot implementation. To clarify this, we revised Table 1 (title and legend) to explicitly state that the consultation did not include formal prioritization or ranking of barriers or solutions. We also expanded the limitations section to acknowledge the absence of formal prioritization methods and discuss the implications for interpretation of the findings.

We also agree that the process used to derive the ten key implementation considerations required further clarification. To address this, we expanded the Methods section to describe how stakeholder inputs from discussion notes, completed worksheets, Mentimeter® responses, and field notes were collated and synthesized. We further clarified that recurring implementation considerations were identified through areas of convergence across multiple consultation activities, including discussions on proposed solutions, pilot procedures, workflow design, assessment tools, training requirements, and site readiness. Corresponding revisions were also made to the Results section to explain how the key implementation considerations emerged from recurring issues identified across these consultation activities and subsequently informed refinement of the care pathway, workflow, assessment tools, training program, and implementation plan.

**Comment 6:** Some conclusions appear overstated. For example, the manuscript describes the model as “feasible,” although feasibility was not formally evaluated through implementation outcomes such as uptake, retention, fidelity, or cost. It would be more accurate to describe the model as “perceived to be feasible by participating stakeholders.” Similarly, statements such as “stakeholders agreed” should be supported with clearer indication of the level of agreement.

**Response:** Thank you for this comment. We agree that feasibility was not formally evaluated in this stakeholder consultation and that implementation outcomes such as feasibility, acceptability, adoption, fidelity, retention, and cost will need to be assessed during pilot implementation. Accordingly, we revised the manuscript to avoid implying demonstrated implementation success and to distinguish stakeholder recommendations and perceptions from formally evaluated implementation outcomes. We also clarified that future pilot implementation will be necessary to evaluate implementation outcomes and determine the effectiveness of the stakeholder-informed approaches in practice.

With regard to stakeholder agreement, the consultation did not employ formal consensus methods or quantitative measures of agreement. Rather, recommendations were refined through facilitated discussion, and areas of agreement were identified through stakeholder deliberation and confirmation during the consultation. We have clarified this process in the Methods section and revised wording where appropriate.

**Comment 7:** The consultation was conducted in April 2023, but the manuscript was submitted several years later. Although the HIV self-testing guideline update is mentioned, the discussion should better address other important developments since 2023, including updates in Malaysian HIV guidelines and regional developments in long-acting injectable PrEP. A short section discussing the current relevance of the recommendations would strengthen the paper.

**Response:** Thank you for this comment. We agree that developments in HIV prevention since the 2023 consultation warrant consideration. To address this, we added a short paragraph to the Discussion acknowledging recent developments in Malaysia, including publication of national guidelines in 2025 to support implementation of the national PrEP programme and the evolving landscape of HIV prevention.

We also acknowledge growing interest in alternative PrEP modalities, including long-acting injectable PrEP. Recent implementation research on pharmacy-based delivery of long-acting injectable antiretrovirals has identified similar considerations related to privacy and confidentiality, workforce training, workflow integration, and collaboration between healthcare providers, suggesting that many of the implementation considerations identified through this consultation remain relevant as PrEP delivery continues to evolve. As the consultation was intended to inform pilot implementation of an oral PrEP service model within community pharmacies, we limited discussion of emerging PrEP modalities to maintain the focus and scope of the manuscript.

Additional references added:

- Ministry of Health Malaysia. National guidelines on HIV PrEP program implementation 2025. Putrajaya: Ministry of Health Malaysia; 2025.
- Chong N, Chan C, Tan Tze Tho A, Schmidt HA, Ong JJ, Tan JFJ, et al. Preferences for emerging modalities of HIV pre-exposure prophylaxis among sexual and gender diverse populations in Malaysia: implications for person-centred HIV prevention. Sex Health. 2026;23(2):SH25092. doi:10.1071/SH25092
- Cocohoba J, Cuca Y, Sherman E, Hester K, Udeani G, Sigua M, et al. Preparing for pharmacy-based delivery of long-acting injectable antiretrovirals: a pre-implementation study. BMC Health Serv Res. 2025;25(1):808. doi:10.1186/s12913-025-12971-8

**Comment 8:** Some important implementation issues are discussed without supporting quantitative information. For example:

- No estimate of private-sector PrEP cost is provided despite affordability being a central issue
- Pharmacist remuneration or opportunity cost is discussed without financial estimates
- The figures describing the number of PrEP facilities may need updating if newer national data are available.

**Response:** We agree that affordability, profitability, and opportunity costs are important considerations for pharmacy-led PrEP implementation. Affordability and profitability/opportunity costs were identified as barriers during the preceding exploratory phase and were subsequently discussed during the stakeholder consultation to identify potential solutions.

To provide additional context regarding affordability, we strengthened the Discussion by incorporating available evidence on willingness-to-pay among Malaysian MSM and relating this to the estimated monthly cost of the pilot service. We also added contextual information regarding the provision of professional community pharmacy services in Malaysia, where many services are delivered without a formal remuneration mechanism, to help explain stakeholder concerns regarding profitability and opportunity costs. Quantification of pharmacist remuneration, profitability, or opportunity costs was beyond the scope of this study, and these data were not collected. Dedicated economic evaluation would be required to assess these considerations.

Additional references added:

- Hussain R, Babar ZU. Global landscape of community pharmacy services remuneration: a narrative synthesis of the literature. J Pharm Policy Pract. 2023;16(1):118. doi:10.1186/s40545-023-00626-0

We also reviewed the figures describing PrEP service availability against the latest available national reports. The 2025 Malaysian Global AIDS Monitoring report did not provide updated figures beyond those reported in the 2024 Malaysian Global AIDS Monitoring report. Therefore, the figures cited in the manuscript were retained.

**Comment 9:** The discussion becomes repetitive because many paragraphs follow a similar structure, where stakeholder concerns are compared with findings from Kenya or Nigeria before being linked back to Malaysia. The section would read more clearly if reorganised around broader implementation themes such as regulatory barriers, financing, workforce readiness, and equity-related challenges.

**Response:** We agree that several sections of the Discussion followed a similar structure when comparing our findings with previous pharmacy-led PrEP studies from Kenya and Nigeria. We reviewed the Discussion and revised the text to reduce repetition, improve flow, and strengthen links across related implementation considerations. We also streamlined comparisons with previous studies and placed greater emphasis on the implications of the findings for implementation within the Malaysian context.

# Response to Reviewer #2:

Reviewer #2: This manuscript presents a stakeholder-informed pre-implementation consultation to design a pharmacy-led PrEP service delivery model in Malaysia. The topic is highly relevant, and the study addresses an important implementation gap in HIV prevention access. The paper is generally well written and operationally rich, but it would benefit from clearer methodological transparency, more disciplined prioritization of findings, and a more cautious interpretation of feasibility.

**Comment 1 (Major):** Clarify how stakeholder input was synthesized. The manuscript states that inputs were synthesized descriptively rather than using formal qualitative coding or thematic analysis, but the process is still too vague for readers to assess rigor.

Suggest: Please specify who reviewed the worksheets and field notes, how convergence and divergence were identified, and whether any structured framework was used to determine which ideas became “key implementation considerations.” A short description of any reconciliation process between reviewers would improve transparency.

**Response:** We agree that greater transparency regarding the synthesis process would strengthen the manuscript. To address this, we expanded the Methods section to provide additional detail on how stakeholder inputs were reviewed and synthesized.

Specifically, we clarified that inputs from discussion notes, completed worksheets, and Mentimeter® responses were collated, reviewed, and synthesized descriptively by two members of the research team (YG and SS). We further clarified that areas of convergence were identified when similar issues, recommendations, or implementation needs were raised across multiple consultation activities, including discussions on proposed solutions, workflow design, pilot procedures, assessment tools, training requirements, and site readiness. Any differences in interpretation were discussed and resolved through consensus prior to finalization of the synthesized findings.

We also clarified that no structured framework, scoring system, or formal prioritization method was used to determine the key implementation considerations. Rather, these considerations emerged through descriptive synthesis of recurring issues raised across multiple consultation activities and stakeholder groups. As the consultation was intended to support implementation planning rather than generate formal qualitative findings, no qualitative coding framework or thematic analysis approach was applied.

To enhance transparency, we additionally described review of the synthesized findings by YG, SS, and a senior clinician-researcher with expertise in HIV prevention (IA), as well as stakeholder validation of the synthesized findings through circulation of a summary report for feedback and accuracy checking.

**Comment 2 (Major):** Explain the rationale for stakeholder selection and potential bias.
The stakeholder group appears diverse, but it is also largely composed of actors already involved in or supportive of the pilot, including implementation partners and service-affiliated stakeholders. This may have favored feasibility-oriented responses and limited critical viewpoints.

Suggest: The authors should clarify the selection logic, note any stakeholder groups not represented, and more explicitly acknowledge how this may have shaped the recommendations.

**Response:** Thank you for this comment. We agree that greater clarity regarding stakeholder selection and potential sources of bias would strengthen the manuscript. To address this, we expanded the Methods section to clarify stakeholder selection, including the criteria used to identify stakeholders with direct roles in PrEP delivery, clinical care, community engagement, or implementation, as well as the rationale for selecting the participating implementation partners.

We also strengthened the Limitations section to acknowledge that the private pharmacy and telemedicine stakeholders represented a selective group already committed to the pilot study. We further clarified that this may have limited the diversity of perspectives and influenced recommendations toward approaches considered acceptable or practical within the participating organizations, while perspectives from stakeholders with more conservative views on pharmacist roles or differing regulatory perspectives may have been underrepresented.

In addition, we clarified that the consultation included only one community pharmacy organization and one telemedicine provider and that representatives from the MOH Disease Control Division and Pharmacy Programme did not participate in their official programmatic or regulatory capacities. We therefore acknowledge that the findings may not fully reflect perspectives of all community pharmacy settings, telemedicine providers, or regulatory stakeholders, particularly in settings without established telemedicine and physician-prescribing partnerships.

**Comment 3 (Major):** Address the lack of prioritization. The consultation generated many useful recommendations, but there is no formal ranking of barriers or strategies. As a result, it is difficult to determine which items were viewed as most important for immediate implementation versus longer-term scale-up.

Suggest: The manuscript should more clearly distinguish core pilot requirements from aspirational system-level reforms, and ideally state that prioritization was not performed.

**Response:** Thank you for this comment. To address this, we clarified in both the Results and Limitations sections that no formal prioritization, scoring system, or structured framework was used to rank barriers, implementation strategies, or key implementation considerations. Rather, the identified considerations emerged through descriptive synthesis of recurring issues raised across multiple consultation activities and stakeholder groups.

We also clarified the distinction between recommendations that informed pilot implementation and those representing longer-term system-level considerations. Most short-term solutions identified during the consultation were incorporated into the pilot procedures, care pathway, workflow, assessment tools, training programme, and implementation plan. Exceptions included the proposed use of pre-visit online risk assessments and telepharmacy with medication delivery options, which were not incorporated into the pilot because stakeholders perceived that an in-person service model would be more practical during the initial implementation phase. In contrast, several longer-term recommendations, including regulatory, policy, and financing reforms, were identified as important considerations for future scale-up of pharmacy-led PrEP services and would require broader system-level changes.

**Comment 4 (Minor):** The Discussion would benefit from a more explicit comparison with the Kenya and Nigeria stakeholder consultation models, since those are cited as important comparators throughout the manuscript.

**Response:** We have added a short paragraph to the Discussion that more explicitly compares the findings of this stakeholder consultation with those reported from similar consultations conducted in Kenya and Nigeria. We highlight several common implementation considerations identified across settings, including privacy and confidentiality, affordability, workforce training, workflow design, and cross-sector collaboration, while also noting context-specific considerations related to Malaysia’s regulatory environment, physician prescribing requirements, telemedicine partnerships, and HIV self-testing regulations. This comparison helps contextualize our findings within the broader literature on pharmacy-led PrEP implementation and highlights the importance of local adaptation when implementing pharmacy-led PrEP services.

In summary, this is a promising and policy-relevant implementation manuscript with practical value for PrEP scale-up, but the current version needs more methodological clarity and a more restrained conclusion.

6. PLOS authors have the option to publish the peer review history of their article ([what does this mean?](https://track.editorialmanager.com/CL0/https:%2F%2Fjournals.plos.org%2Fplosone%2Fs%2Feditorial-and-peer-review-process%23loc-peer-review-history/1/010f019e5d29b399-4b86f61a-2b50-4bc3-bfc9-ce9afbe774e8-000000/DXl8wtFd98tKxhO6hVWAJo6_YQS_tlh-USJieku60iA=258)). If published, this will include your full peer review and any attached files.

If you choose “no”, your identity will remain anonymous but your review may still be made public.

**Do you want your identity to be public for this peer review?** For information about this choice, including consent withdrawal, please see our [Privacy Policy](https://track.editorialmanager.com/CL0/https:%2F%2Fwww.plos.org%2Fprivacy-policy/1/010f019e5d29b399-4b86f61a-2b50-4bc3-bfc9-ce9afbe774e8-000000/O9pwpXdcUO9dB8qjOtiHU55-t3C1i4KUnOmjtHeO2s0=258).

Reviewer #1: No

Reviewer #2: No

We hope that our revisions adequately address the concerns raised. Please let us know if further modifications are required. Once again, we appreciate the editor’s and reviewers’ valuable feedback, which has helped us improve our manuscript.

Thank you for your time and consideration.

Best regards,

**Professor Frederick L. Altice**

Professor of Medicine, Epidemiology, and Public Health

Yale School of Medicine

Yale University, United States of America

frederick.altice@yale.edu
