# Supplementary material for: Stakeholder-informed pre-implementation refinement of a pharmacy-led pre-exposure prophylaxis (PrEP) service delivery model in Malaysia
Source: PLoS One. 2026 Sep 8;21(9):e0357920. doi: 10.1371/journal.pone.0357920 (PMC13552737; doi:10.1371/journal.pone.0357920)
Supplement: Supplementary file 8 [file pone.0357920.s009.docx]

**Professor Frederick L. Altice**

Professor of Medicine, Epidemiology, and Public Health

Yale School of Medicine

Yale University, United States of America

frederick.altice@yale.edu

5^th^ August 2026

**Academic Editor
PLOS ONE**

**Subject: Response to Reviewers for Manuscript Revision (PONE-D-26-19386R1)**

Dear Dr. Ali Haider Mohammed,

We sincerely appreciate the opportunity to revise our manuscript, titled "Stakeholder-informed pre-implementation refinement of a pharmacy-led pre-exposure prophylaxis (PrEP) service delivery model in Malaysia" (Manuscript ID: PONE-D-26-19386R1).

We are grateful for the constructive feedback provided by the academic editor and reviewers. We have carefully addressed each comment and have made the necessary revisions to enhance the clarity and quality of our manuscript. Below, we provide a point-by-point response to each comment, detailing the changes made.

# Journal requirements:

**Comment 1:** If the reviewer comments include a recommendation to cite specific previously published works, please review and evaluate these publications to determine whether they are relevant and should be cited. There is no requirement to cite these works unless the editor has indicated otherwise.

**Response:** We have reviewed and evaluated all reviewer-recommended publications for their relevance to our study. Relevant publications have been incorporated into the revised manuscript where appropriate.

**Comment 2:** Please review your reference list to ensure that it is complete and correct. If you have cited papers that have been retracted, please include the rationale for doing so in the manuscript text, or remove these references and replace them with relevant current references. Any changes to the reference list should be mentioned in the rebuttal letter that accompanies your revised manuscript. If you need to cite a retracted article, indicate the article’s retracted status in the References list and also include a citation and full reference for the retraction notice.

**Response:** We have reviewed the reference list to ensure that all references are complete, accurate, and formatted according to the journal requirements. We also verified that none of the cited publications are retracted. Minor corrections have been made to the reference list where appropriate.

# Additional editor comments:

**Comment 1:** Please consider adding a brief table summarizing stakeholder characteristics (stakeholder group, number of participants, and role). This would improve transparency and allow readers to better appreciate the diversity of perspectives included in the consultation.

**Response:** Thank you for this helpful suggestion. We agree that a summary table improves the transparency of the stakeholder consultation. We have added Table 1, which summarizes the stakeholder groups, number of stakeholders, and their roles in the consultation. The table also clarifies overlapping stakeholder roles where applicable. To avoid duplication, we have streamlined the corresponding description in the Methods section and moved the detailed stakeholder information to the table.

**Comment 2:** Table 1 is comprehensive but difficult to navigate. Consider indicating which short-term recommendations were ultimately incorporated into the pilot implementation (e.g., using shading, symbols, or an additional column), making it easier for readers to distinguish implemented versus proposed recommendations.

**Response:** We thank the editor for this helpful suggestion. We agree that distinguishing proposed solutions incorporated into the pilot implementation improves the transparency and readability of the table. Rather than adding a column, we have used a check mark (√) to identify proposed solutions that were incorporated into the pilot pharmacy-led PrEP service model and added an explanatory footnote. We have also clarified in the Methods how stakeholders and the research team determined which proposed short-term solutions would be incorporated into the pilot implementation. Finally, we streamlined the wording of several proposed solutions to improve readability and conciseness while preserving the intent of the stakeholder-generated solutions.

**Comment 3:** The description of the pharmacist training programme could be strengthened by briefly stating the anticipated delivery format (e.g., online modules, workshops, simulation exercises) or expected duration, even if detailed information is provided in the supplementary materials.

**Response:** We appreciate this suggestion. To provide additional context while maintaining the focus of the manuscript on the stakeholder consultation, we have expanded the Discussion to briefly describe the pharmacist PrEP training programme that was subsequently developed based on the stakeholder recommendations. Specifically, we now state that the programme comprised self-paced online learning followed by an in-person workshop to support pilot implementation, and we refer readers to our previously published paper describing the development and evaluation of the online training component [Ref. 30].

**Comment 4:** The Discussion could be strengthened by briefly commenting on the applicability of the proposed implementation model to other middle-income countries in Southeast Asia, as this would enhance the broader relevance of the findings beyond Malaysia.

**Response:** Thank you for this valuable suggestion. We agree that discussing the broader applicability of the proposed implementation model enhances the relevance of our findings beyond the Malaysian context. We have therefore expanded the Discussion to comment on the potential applicability of the model to other middle-income countries in Southeast Asia, while emphasising that transferability will depend on country-specific regulatory frameworks, healthcare financing, pharmacy infrastructure, and the availability of laboratory and referral services. We also clarify that the Malaysian model should be viewed as a contextually adaptable implementation approach rather than a directly transferable service model.

**Comment 5:** Please consider acknowledging that implementation priorities identified during the consultation may evolve over time as national HIV policies, PrEP delivery models, and regulatory frameworks continue to develop.

**Response:** Thank you for highlighting this important point. We agree that acknowledging the evolving implementation landscape strengthens the broader applicability of our findings. We have therefore expanded the Discussion to acknowledge that the implementation considerations identified through this stakeholder consultation reflect the policy, regulatory, and service delivery context at the time of the consultation. We also note that their relative relevance may evolve as national HIV policies, PrEP delivery models, prescribing arrangements, and regulatory frameworks continue to develop, and that periodic stakeholder reassessment will be important to ensure that the service delivery model remains responsive to changing healthcare needs and implementation contexts.

**Comment 6:** The text within Figures 1–3 appears relatively small and may be difficult to read in the final published PDF. Increasing the font size would improve figure readability.

**Response:** Thank you for highlighting this. We have revised Figures 1–3 by increasing the font size and making minor layout adjustments where necessary to improve readability in the final published PDF.

**Reviewer’s comments:**

**Reviewer's Responses to Questions**

**Comments to the Author**

1. If the authors have adequately addressed your comments raised in a previous round of review and you feel that this manuscript is now acceptable for publication, you may indicate that here to bypass the “Comments to the Author” section, enter your conflict of interest statement in the “Confidential to Editor” section, and submit your "Accept" recommendation.

Reviewer #1: All comments have been addressed

Reviewer #2: All comments have been addressed

2. Is the manuscript technically sound, and do the data support the conclusions?

The manuscript must describe a technically sound piece of scientific research with data that supports the conclusions. Experiments must have been conducted rigorously, with appropriate controls, replication, and sample sizes. The conclusions must be drawn appropriately based on the data presented.

Reviewer #1: Yes

Reviewer #2: Yes

3. Has the statistical analysis been performed appropriately and rigorously?

Reviewer #1: Yes

Reviewer #2: Yes

4. Have the authors made all data underlying the findings in their manuscript fully available?

The [PLOS Data policy](http://track.editorialmanager.com/CL0/http:%2F%2Fwww.plosone.org%2Fstatic%2Fpolicies.action%23sharing/1/010f019fa1ecce76-9461d1ae-d1f5-4f03-9986-6b7e7d3932ed-000000/h4oOcF8y7Ugcv3tmt-8RHyRfE1yhVcxsxuqbkkLcisE=258) requires authors to make all data underlying the findings described in their manuscript fully available without restriction, with rare exception (please refer to the Data Availability Statement in the manuscript PDF file). The data should be provided as part of the manuscript or its supporting information, or deposited to a public repository. For example, in addition to summary statistics, the data points behind means, medians and variance measures should be available. If there are restrictions on publicly sharing data—e.g. participant privacy or use of data from a third party—those must be specified.

Reviewer #1: Yes

Reviewer #2: Yes

5. Is the manuscript presented in an intelligible fashion and written in standard English?

PLOS ONE does not copyedit accepted manuscripts, so the language in submitted articles must be clear, correct, and unambiguous. Any typographical or grammatical errors should be corrected at revision, so please note any specific errors here.

Reviewer #1: Yes

Reviewer #2: Yes

**6. Review Comments to the Author**

# Response to Reviewer #1:

Reviewer #1: Thank you to the authors for their thorough revision. The manuscript has improved substantially, and my major concerns have been addressed.

The revised Methods and limitations now clearly explain the involvement of community-based organisations and the limited participation of end users. The clarification that Ministry of Health specialists attended as prescribers, rather than in a programmatic or regulatory role, also resolves the earlier inconsistency.

The revised feasibility language is now appropriately cautious, describing the model as perceived by stakeholders to be feasible. It is also helpful that the Results clarify that no formal prioritisation or scoring was used.

**Comment 1:** The new discussion of affordability is particularly valuable. The estimated monthly cost of RM157 is higher than the amount many Malaysian men who have sex with men are willing to pay, with more than half preferring a cost below RM100. The authors may wish to mention this gap briefly in the abstract, as it could affect the model’s sustainability.

**Response:** We thank the reviewer for this helpful suggestion. We agree that affordability is important to the sustainability of pharmacy-led PrEP delivery. After consideration, we retained the cost comparison in the Discussion rather than adding it to the Abstract because the willingness-to-pay estimate was derived from a separate Malaysian study using data collected in 2016. We have expanded the Discussion to clarify the components of the estimated service cost and to note that direct comparison should be interpreted cautiously given changes in economic conditions, PrEP awareness and healthcare financing over time.

I have two minor suggestions:

**Comment 2:** The Methods state that a summary report was circulated to stakeholders for validation. Please consider reporting how many stakeholders responded and whether their feedback led to any substantive changes.

**Response:** Thank you for this helpful suggestion. We have clarified the outcome of the stakeholder feedback process in the Methods section. A summary report was circulated to all 28 stakeholders for feedback and accuracy checking. One stakeholder acknowledged receipt and did not request changes; no other stakeholders responded. Because of the limited response, this circulation was not interpreted as formal stakeholder validation. The manuscript has been revised accordingly.

**Comment 3:** Although a formal qualitative reporting framework was not used, the authors could consider providing the five-item GRIPP2-SF checklist as supporting information if the editor finds it useful.

**Response:** We thank the reviewer for this thoughtful suggestion. We carefully considered providing the GRIPP2-SF checklist. However, we concluded that GRIPP2-SF was not the most appropriate reporting guideline for this study. The stakeholder consultation was conducted to refine the implementation strategy for the pharmacy-led PrEP service by obtaining input from multiple stakeholder groups, including community pharmacists, PrEP prescribers, NGO representatives, telemedicine providers, professional societies, and implementation science researchers. Although NGO representatives participated in the consultation, it was designed as a multi-stakeholder implementation consultation, and participants contributed primarily in their stakeholder roles to inform implementation planning rather than as patients or members of the public acting as partners in the research process. Therefore, we concluded that including the GRIPP2-SF checklist could inadvertently misrepresent the nature of the stakeholder consultation. We have clarified this distinction in the manuscript by describing the stakeholder consultation as a multi-stakeholder consultation conducted to refine the implementation strategy.

# Response to Reviewer #2:

**Comment 1:** Clarify why GRIPP2-SF/SRQR/COREQ were not applicable.

>Suggestion: Given that the consultation was conducted as an implementation-informed stakeholder engagement activity rather than a qualitative research study involving formal qualitative data collection and analysis, we considered SRQR and COREQ not directly applicable; similarly, GRIPP2-SF was not adopted because the activity was not designed as a patient and public involvement initiative.

**Response:** We agree. Thank you.

**Comment 2:** Ensure every use of "feasible," "agreed," "prioritized," and "acceptable" is appropriately qualified.

**Response:** Thank you for this important comment. We have carefully reviewed the manuscript and revised the wording throughout to avoid unqualified use of terms such as "feasible," "agreed," "prioritized," and "acceptable." Where appropriate, we have replaced these terms with more precise language that accurately reflects the stakeholder consultation findings and avoids implying objective conclusions or unanimous consensus.

**Comment 3:** Clarify exactly what the RM157/month estimated cost includes. Does this cover:

PrEP medication? Consultation? HIV testing? Renal function testing? Pharmacy service fee? Telemedicine fee?

**Response:** Thank you for highlighting this. We have clarified in the Discussion that the estimated monthly cost of approximately RM157 included the cost of PrEP medication (RM97) and an HIV self-test kit (RM60) provided through the pharmacy during the study period. We also clarified that medication rebates were introduced during the pilot to reduce financial barriers, allowing participants to access PrEP medication at RM65 per month while HIV self-testing was provided at no cost. For clarity, the reported estimate did not include renal function testing, as this was performed according to clinical need rather than at every visit. Consultation fees were not charged in Malaysian community pharmacies, and telemedicine costs were absorbed by participating pharmacies through their subscription arrangements with the telemedicine provider.

**Comment 4:** Define more explicitly how the 10 key implementation considerations were derived or consider explicitly stating that the ten considerations are researcher-derived synthesis categories rather than formally validated qualitative themes.

**Response:** Thank you for this important comment. We agree that the derivation of the ten implementation considerations should be described more explicitly. We have revised the manuscript to clarify that the ten implementation considerations were researcher-derived through descriptive synthesis of recurring stakeholder inputs across the consultation activities. We have also clarified that these considerations were used as descriptive categories to summarise recurrent implementation issues and recommendations, rather than formally coded qualitative themes or stakeholder-ranked priorities.

7. PLOS authors have the option to publish the peer review history of their article (what does this mean?). If published, this will include your full peer review and any attached files.

If you choose “no”, your identity will remain anonymous but your review may still be made public.

Do you want your identity to be public for this peer review? For information about this choice, including consent withdrawal, please see our Privacy Policy.

Reviewer #1: Yes: Fahmi Hassan

Reviewer #2: No

We hope that our revisions adequately address the concerns raised. Please let us know if further modifications are required. Once again, we appreciate the editor’s and reviewers’ valuable feedback, which has helped us improve our manuscript.

Thank you for your time and consideration.

Best regards,

**Professor Frederick L. Altice**

Professor of Medicine, Epidemiology, and Public Health

Yale School of Medicine

Yale University, United States of America

frederick.altice@yale.edu
