# Supplementary material for: Stakeholder-informed pre-implementation refinement of a pharmacy-led pre-exposure prophylaxis (PrEP) service delivery model in Malaysia
Source: PLoS One. 2026 Sep 8;21(9):e0357920. doi: 10.1371/journal.pone.0357920 (PMC13552737; doi:10.1371/journal.pone.0357920)
Supplement: Peer review history

# Peer review: Decision Letter 0

**DOI:** 10.1371/journal.pone.0357920.r001

**Authors:** Ali Haider Mohammed

24 May 2026

Dear Dr. Altice,

Please submit your revised manuscript by Jul 08 2026 11:59PM. If you will need more time than this to complete your revisions, please reply to this message or contact the journal office at plosone@plos.org. When you're ready to submit your revision, log on to https://www.editorialmanager.com/pone/ and select the 'Submissions Needing Revision' folder to locate your manuscript file.

- A letter that responds to each point raised by the academic editor and reviewer(s). You should upload this letter as a separate file labeled 'Response to Reviewers'.
- A marked-up copy of your manuscript that highlights changes made to the original version. You should upload this as a separate file labeled 'Revised Manuscript with Track Changes'.
- An unmarked version of your revised paper without tracked changes. You should upload this as a separate file labeled 'Manuscript'.

If you would like to make changes to your financial disclosure, please include your updated statement in your cover letter. Guidelines for resubmitting your figure files are available below the reviewer comments at the end of this letter.

If applicable, we recommend that you deposit your laboratory protocols in protocols.io to enhance the reproducibility of your results. Protocols.io assigns your protocol its own identifier (DOI) so that it can be cited independently in the future. For instructions see: https://journals.plos.org/plosone/s/submission-guidelines#loc-laboratory-protocols. Additionally, PLOS ONE offers an option for publishing peer-reviewed Lab Protocol articles, which describe protocols hosted on protocols.io. Read more information on sharing protocols at https://plos.org/protocols?utm_medium=editorial-email&utm_source=authorletters&utm_campaign=protocols.

As the corresponding author, your ORCID iD is verified in the submission system and will appear in the published article. PLOS supports the use of ORCID, and we encourage all coauthors to register for an ORCID iD and use it as well. Please encourage your coauthors to verify their ORCID iD within the submission system before final acceptance, as unverified ORCID iDs will not appear in the published article. *Only*  the individual author can complete the verification step; PLOS staff *cannot*  verify ORCID iDs on behalf of authors.

We look forward to receiving your revised manuscript.

Kind regards,

Ali Haider Mohammed

Academic Editor

PLOS One

Journal Requirements:

When submitting your revision, we need you to address these additional requirements.

1. Please ensure that your manuscript meets PLOS ONE's style requirements, including those for file naming. The PLOS ONE style templates can be found at https://journals.plos.org/plosone/s/file?id=wjVg/PLOSOne_formatting_sample_main_body.pdf and https://journals.plos.org/plosone/s/file?id=ba62/PLOSOne_formatting_sample_title_authors_affiliations.pdf

2. Thank you for stating in your Funding Statement:

[This research was funded by the World Health Organization (WHO) (WHO registration: 2023/1363112-0) – Principal Investigators (PI): Iskandar Azwa https://www.who.int/malaysia/ and the Malaysian Implementation Science Training program (MIST) for YG supported by Fogarty International Center, NIDA, NIMH and NICHD (D43 TW-011324 – PI: Frederick L. Altice, Adeeba Kamarulzaman) https://www.fic.nih.gov/.The funders had no role in study design, information collection and analysis, decision to publish, or preparation of the manuscript.].

Please provide an amended statement that declares *all* the funding or sources of support (whether external or internal to your organization) received during this study, as detailed online in our guide for authors at http://journals.plos.org/plosone/s/submit-now.  Please also include the statement “There was no additional external funding received for this study.” in your updated Funding Statement.

Please include your amended Funding Statement within your cover letter. We will change the online submission form on your behalf.

3. Thank you for stating the following in the Competing Interests section:

[The authors have declared that no competing interests exist.].

We note that one or more of the authors are employed by a commercial company: The Red Clinic.

1. Please provide an amended Funding Statement declaring this commercial affiliation, as well as a statement regarding the Role of Funders in your study. If the funding organization did not play a role in the study design, data collection and analysis, decision to publish, or preparation of the manuscript and only provided financial support in the form of authors' salaries and/or research materials, please review your statements relating to the author contributions, and ensure you have specifically and accurately indicated the role(s) that these authors had in your study. You can update author roles in the Author Contributions section of the online submission form.

Please also include the following statement within your amended Funding Statement.

“The funder provided support in the form of salaries for authors [insert relevant initials], but did not have any additional role in the study design, data collection and analysis, decision to publish, or preparation of the manuscript. The specific roles of these authors are articulated in the ‘author contributions’ section.”

If your commercial affiliation did play a role in your study, please state and explain this role within your updated Funding Statement.

2. Please also provide an updated Competing Interests Statement declaring this commercial affiliation along with any other relevant declarations relating to employment, consultancy, patents, products in development, or marketed products, etc.

Within your Competing Interests Statement, please confirm that this commercial affiliation does not alter your adherence to all PLOS ONE policies on sharing data and materials by including the following statement: "This does not alter our adherence to PLOS ONE policies on sharing data and materials.” (as detailed online in our guide for authors http://journals.plos.org/plosone/s/competing-interests) . If this adherence statement is not accurate and there are restrictions on sharing of data and/or materials, please state these. Please note that we cannot proceed with consideration of your article until this information has been declared.

Please include both an updated Funding Statement and Competing Interests Statement in your cover letter. We will change the online submission form on your behalf.

4. Please include captions for your Supporting Information files at the end of your manuscript, and update any in-text citations to match accordingly. Please see our Supporting Information guidelines for more information: http://journals.plos.org/plosone/s/supporting-information.

5. We note that Figures S6.1 and S6.2 in your submission contain copyrighted images. All PLOS content is published under the Creative Commons Attribution License (CC BY 4.0), which means that the manuscript, images, and Supporting Information files will be freely available online, and any third party is permitted to access, download, copy, distribute, and use these materials in any way, even commercially, with proper attribution. For more information, see our copyright guidelines: http://journals.plos.org/plosone/s/licenses-and-copyright.

We require you to either (1) present written permission from the copyright holder to publish these figures specifically under the CC BY 4.0 license, or (2) remove the figures from your submission:

1. You may seek permission from the original copyright holder of Figures S6.1 and S6.2 to publish the content specifically under the CC BY 4.0 license.

We recommend that you contact the original copyright holder with the Content Permission Form (http://journals.plos.org/plosone/s/file?id=7c09/content-permission-form.pdf) and the following text:

“I request permission for the open-access journal PLOS ONE to publish XXX under the Creative Commons Attribution License (CCAL) CC BY 4.0 (http://creativecommons.org/licenses/by/4.0/). Please be aware that this license allows unrestricted use and distribution, even commercially, by third parties. Please reply and provide explicit written permission to publish XXX under a CC BY license and complete the attached form.”

Please upload the completed Content Permission Form or other proof of granted permissions as an "Other" file with your submission.

In the figure caption of the copyrighted figure, please include the following text: “Reprinted from [ref] under a CC BY license, with permission from [name of publisher], original copyright [original copyright year].”

2. If you are unable to obtain permission from the original copyright holder to publish these figures under the CC BY 4.0 license or if the copyright holder’s requirements are incompatible with the CC BY 4.0 license, please either i) remove the figure or ii) supply a replacement figure that complies with the CC BY 4.0 license. Please check copyright information on all replacement figures and update the figure caption with source information. If applicable, please specify in the figure caption text when a figure is similar but not identical to the original image and is therefore for illustrative purposes only.

If the reviewer comments include a recommendation to cite specific previously published works, please review and evaluate these publications to determine whether they are relevant and should be cited. There is no requirement to cite these works unless the editor has indicated otherwise.

Additional Editor Comments:

The manuscript repeatedly states the consultation was “not qualitative research” but simultaneously uses discussions, field notes, structured worksheets, descriptive synthesis, and “member checking.”

This creates conceptual confusion.'

Limited analytical depth. The “Results” section is largely descriptive listing of barriers and solutions.

There is:

no prioritization framework, no ranking of implementation barriers, no systematic coding structure, no explicit implementation determinant mapping (e.g., CFIR domains),

limited synthesis beyond narrative summaries.

For implementation science audiences, this weakens rigor.

The discussion repeatedly states phrases like:

“This reflects an implementation determinant…”

This becomes formulaic and reads somewhat AI-assisted/editorially repetitive.

The manuscript could likely be reduced by 20–25% without losing substance.

Potential selection bias

Stakeholders were heavily drawn from implementation partners already committed to the pilot:

Alpro Pharmacy,

DOC2US,

collaborating clinicians/NGOs.

This risks:

confirmation bias,

overly favorable feasibility perceptions,

underrepresentation of dissenting regulatory or conservative pharmacy perspectives.

The limitation section acknowledges this, but only briefly.

I would want deeper critical reflection.

The manuscript sometimes overextends implications for “Malaysia” broadly despite likely limited rural applicability.

Some implementation claims are speculative

Statements about:

scalability,

affordability,

sustainability,

public-private reimbursement,

workflow feasibility

are not empirically tested here. They are stakeholder perceptions.

The authors should more explicitly distinguish:

stakeholder opinion,

vs

demonstrated implementation outcomes.

[Note: HTML markup is below. Please do not edit.]

Reviewers' comments:

Reviewer's Responses to Questions

**Comments to the Author**

1. Is the manuscript technically sound, and do the data support the conclusions?

Reviewer #1: Partly

Reviewer #2: Yes

**********

2. Has the statistical analysis been performed appropriately and rigorously?

Reviewer #1: N/A

Reviewer #2: N/A

**********

3. Have the authors made all data underlying the findings in their manuscript fully available?

The PLOS Data policy

Reviewer #1: Yes

Reviewer #2: Yes

**********

4. Is the manuscript presented in an intelligible fashion and written in standard English?

Reviewer #1: Yes

Reviewer #2: Yes

**********

Reviewer #1: 1. The proposed service targets key populations such as MSM, transgender women, and people who inject drugs, yet none of the consultation participants directly represented these groups. Although the authors referred to prior formative interviews, the consultation itself was largely provider- and system-focused. Therefore, conclusions related to privacy, stigma, acceptability, and service preferences should be interpreted cautiously. This limitation should be stated more explicitly in the manuscript.

2. Several major barriers identified in the manuscript, including prescribing restrictions, Group B Poison classification of TDF/FTC, HIV testing regulations, and reimbursement policies, fall directly under MOH authority. Many proposed long-term solutions in Table 1 would require involvement from the Disease Control Division and Pharmacy Programme. In addition, there appears to be inconsistency between the methods section, which states MOH representatives were not invited, and the acknowledgements section listing MOH specialists. This should be clarified.

3. Only one pharmacy chain and one telemedicine provider participated in the consultation. As a result, the proposed workflow and operational recommendations may reflect the systems and resources already available within these organisations. Independent pharmacies and smaller chains may face different challenges. The authors should avoid overgeneralising the findings to all Malaysian community pharmacies.

4. The manuscript states that stakeholder inputs were synthesised descriptively without formal qualitative analysis. However, the paper still presents thematic and interpretive conclusions, which creates some methodological inconsistency. Additional details are needed, including:

* facilitator roles and positioning

* whether discussions were audio-recorded or only field-noted

* how convergence of ideas was determined

* how disagreements were managed

* the process and outcomes of member checking

The authors may also consider using a reporting framework such as GRIPP2-SF, SRQR, or COREQ to improve transparency.

5. Table 1 contains many suggested solutions, but no formal prioritisation method was applied. Therefore, the findings only show that stakeholders identified these issues, not which were considered most important or most feasible. The process used to derive the ten “key implementation considerations” in Figure 3 should also be described more clearly.

6. Some conclusions appear overstated. For example, the manuscript describes the model as “feasible,” although feasibility was not formally evaluated through implementation outcomes such as uptake, retention, fidelity, or cost. It would be more accurate to describe the model as “perceived to be feasible by participating stakeholders.” Similarly, statements such as “stakeholders agreed” should be supported with clearer indication of the level of agreement.

7. The consultation was conducted in April 2023, but the manuscript was submitted several years later. Although the HIV self-testing guideline update is mentioned, the discussion should better address other important developments since 2023, including updates in Malaysian HIV guidelines and regional developments in long-acting injectable PrEP. A short section discussing the current relevance of the recommendations would strengthen the paper.

8. Some important implementation issues are discussed without supporting quantitative information. For example:

* no estimate of private-sector PrEP cost is provided despite affordability being a central issue

* pharmacist remuneration or opportunity cost is discussed without financial estimates

* the figures describing the number of PrEP facilities may need updating if newer national data are available

9. The discussion becomes repetitive because many paragraphs follow a similar structure, where stakeholder concerns are compared with findings from Kenya or Nigeria before being linked back to Malaysia. The section would read more clearly if reorganised around broader implementation themes such as regulatory barriers, financing, workforce readiness, and equity-related challenges.

Reviewer #2: This manuscript presents a stakeholder-informed pre-implementation consultation to design a pharmacy-led PrEP service delivery model in Malaysia. The topic is highly relevant, and the study addresses an important implementation gap in HIV prevention access. The paper is generally well written and operationally rich, but it would benefit from clearer methodological transparency, more disciplined prioritization of findings, and a more cautious interpretation of feasibility.

Major comments:

1) Clarify how stakeholder input was synthesized.

The manuscript states that inputs were synthesized descriptively rather than using formal qualitative coding or thematic analysis, but the process is still too vague for readers to assess rigor.

Suggest: Please specify who reviewed the worksheets and field notes, how convergence and divergence were identified, and whether any structured framework was used to determine which ideas became “key implementation considerations.” A short description of any reconciliation process between reviewers would improve transparency

2) Explain the rationale for stakeholder selection and potential bias.

The stakeholder group appears diverse, but it is also largely composed of actors already involved in or supportive of the pilot, including implementation partners and service-affiliated stakeholders. This may have favored feasibility-oriented responses and limited critical viewpoints.

Suggest: The authors should clarify the selection logic, note any stakeholder groups not represented, and more explicitly acknowledge how this may have shaped the recommendations.

3) Address the lack of prioritization.

The consultation generated many useful recommendations, but there is no formal ranking of barriers or strategies. As a result, it is difficult to determine which items were viewed as most important for immediate implementation versus longer-term scale-up.

Suggest: The manuscript should more clearly distinguish core pilot requirements from aspirational system-level reforms, and ideally state that prioritization was not performed.

Minor comment:

1) The Discussion would benefit from a more explicit comparison with the Kenya and Nigeria stakeholder consultation models, since those are cited as important comparators throughout the manuscript.

In summary, this is a promising and policy-relevant implementation manuscript with practical value for PrEP scale-up, but the current version needs more methodological clarity and a more restrained conclusion.

**********

what does this mean?). If published, this will include your full peer review and any attached files.

If you choose “no”, your identity will remain anonymous but your review may still be made public.

**Do you want your identity to be public for this peer review?**  For information about this choice, including consent withdrawal, please see our Privacy Policy

Reviewer #1: No

Reviewer #2: No

**********

[NOTE: If reviewer comments were submitted as an attachment file, they will be attached to this email and accessible via the submission site. Please log into your account, locate the manuscript record, and check for the action link "View Attachments". If this link does not appear, there are no attachment files.]

To ensure your figures meet our technical requirements, please review our figure guidelines: https://journals.plos.org/plosone/s/figures

You may also use PLOS’s free figure tool, NAAS, to help you prepare publication quality figures: https://journals.plos.org/plosone/s/figures#loc-tools-for-figure-preparation.

NAAS will assess whether your figures meet our technical requirements by comparing each figure against our figure specifications.

# Author comment: Author response to Decision Letter 1

**DOI:** 10.1371/journal.pone.0357920.r002

3 Jul 2026

Professor Frederick L. Altice

Professor of Medicine, Epidemiology, and Public Health

Yale School of Medicine

Yale University, United States of America

frederick.altice@yale.edu

25th June 2026

Editor-in-Chief

PLOS ONE

Subject: Response to Reviewers for Manuscript Revision (PONE-D-26-19386)

Dear Dr. Ali Haider Mohammed,

We sincerely appreciate the opportunity to revise our manuscript, titled "Stakeholder-informed pre-implementation design of a pharmacy-led pre-exposure prophylaxis (PrEP) service delivery model in Malaysia" (Manuscript ID: PONE-D-26-19386).

We are grateful for the constructive feedback provided by the academic editor and reviewers. We have carefully addressed each comment and have made the necessary revisions to enhance the clarity and quality of our manuscript. Below, we provide a point-by-point response to each comment, detailing the changes made.

________________________________________

Journal requirements:

Comment 1: Please ensure that your manuscript meets PLOS ONE's style requirements, including those for file naming. The PLOS ONE style templates can be found at https://journals.plos.org/plosone/s/file?id=wjVg/PLOSOne_formatting_sample_main_body.pdf and https://journals.plos.org/plosone/s/file?id=ba62/PLOSOne_formatting_sample_title_authors_affiliations.pdf

Response: Thank you for highlighting the journal formatting requirements. We have carefully revised the manuscript to ensure compliance with PLOS ONE style requirements, including formatting, author affiliations, corresponding author formatting, supporting information labeling, and file naming conventions, in accordance with the journal guidelines and templates.

Comment 2: Thank you for stating in your Funding Statement:

[This research was funded by the World Health Organization (WHO) (WHO registration: 2023/1363112-0) – Principal Investigators (PI): Iskandar Azwa https://www.who.int/malaysia/ and the Malaysian Implementation Science Training program (MIST) for YG supported by Fogarty International Center, NIDA, NIMH and NICHD (D43 TW-011324 – PI: Frederick L. Altice, Adeeba Kamarulzaman) https://www.fic.nih.gov/.The funders had no role in study design, information collection and analysis, decision to publish, or preparation of the manuscript.].

Please provide an amended statement that declares *all* the funding or sources of support (whether external or internal to your organization) received during this study, as detailed online in our guide for authors at http://journals.plos.org/plosone/s/submit-now. Please also include the statement “There was no additional external funding received for this study.” in your updated Funding Statement.

Please include your amended Funding Statement within your cover letter. We will change the online submission form on your behalf.

Response: We have revised the Funding Statement to include all sources of funding and support received for this study. The revised Funding Statement is as follows:

“This activity was funded by the World Health Organization (WHO) (WHO registration: 2023/1363112-0; Principal Investigator: Iskandar Azwa) https://www.who.int/malaysia/ and the Malaysian Implementation Science Training program (MIST) for YG, supported by the Fogarty International Center, NIDA, NIMH, and NICHD (D43 TW-011324; Principal Investigators: Frederick L. Altice and Adeeba Kamarulzaman) https://www.fic.nih.gov/. The funders had no role in study design, information collection and analysis, decision to publish, or preparation of the manuscript. There was no additional external funding received for this study. The Red Clinic provided salary support to author AY in the form of employment but did not provide funding for this study and had no additional role in study design, information collection and analysis, decision to publish, or preparation of the manuscript. The specific role of this author is articulated in the ‘author contributions’ section.”

We have included the amended Funding Statement in our cover letter.

Comment 3: Thank you for stating the following in the Competing Interests section:

[The authors have declared that no competing interests exist.].

We note that one or more of the authors are employed by a commercial company: The Red Clinic.

1. Please provide an amended Funding Statement declaring this commercial affiliation, as well as a statement regarding the Role of Funders in your study. If the funding organization did not play a role in the study design, data collection and analysis, decision to publish, or preparation of the manuscript and only provided financial support in the form of authors' salaries and/or research materials, please review your statements relating to the author contributions, and ensure you have specifically and accurately indicated the role(s) that these authors had in your study. You can update author roles in the Author Contributions section of the online submission form.

Please also include the following statement within your amended Funding Statement.

“The funder provided support in the form of salaries for authors [insert relevant initials], but did not have any additional role in the study design, data collection and analysis, decision to publish, or preparation of the manuscript. The specific roles of these authors are articulated in the ‘author contributions’ section.”

If your commercial affiliation did play a role in your study, please state and explain this role within your updated Funding Statement.

2. Please also provide an updated Competing Interests Statement declaring this commercial affiliation along with any other relevant declarations relating to employment, consultancy, patents, products in development, or marketed products, etc.

Within your Competing Interests Statement, please confirm that this commercial affiliation does not alter your adherence to all PLOS ONE policies on sharing data and materials by including the following statement: "This does not alter our adherence to PLOS ONE policies on sharing data and materials.” (as detailed online in our guide for authors http://journals.plos.org/plosone/s/competing-interests) . If this adherence statement is not accurate and there are restrictions on sharing of data and/or materials, please state these. Please note that we cannot proceed with consideration of your article until this information has been declared.

Please include both an updated Funding Statement and Competing Interests Statement in your cover letter. We will change the online submission form on your behalf.

Response: We have updated both the Funding Statement and Competing Interests Statement in our cover letter to clarify the commercial affiliation identified by the journal.

The revised Competing Interests Statement is as follows:

“AY is affiliated with The Red Clinic, a private healthcare provider in Malaysia, and participated in this study in his professional capacity as a private-sector PrEP prescriber and committee member of the professional society Malaysian Society for HIV Medicine (MASHM). The Red Clinic provided salary support to author AY in the form of employment but did not provide funding for this study and had no additional role in study design, information collection and analysis, decision to publish, or preparation of the manuscript. The remaining authors declare that no competing interests exist. This does not alter our adherence to PLOS ONE policies on sharing data and materials.”

Comment 4: Please include captions for your Supporting Information files at the end of your manuscript, and update any in-text citations to match accordingly. Please see our Supporting Information guidelines for more information: http://journals.plos.org/plosone/s/supporting-information.

Response: We have reviewed the Supporting Information files, captions, file names, and corresponding in-text citations to ensure consistency with PLOS ONE Supporting Information guidelines. Supporting Information captions are provided at the end of the manuscript, and all Supporting Information files and citations have been updated accordingly.

Comment 5: We note that Figures S6.1 and S6.2 in your submission contain copyrighted images. All PLOS content is published under the Creative Commons Attribution License (CC BY 4.0), which means that the manuscript, images, and Supporting Information files will be freely available online, and any third party is permitted to access, download, copy, distribute, and use these materials in any way, even commercially, with proper attribution. For more information, see our copyright guidelines: http://journals.plos.org/plosone/s/licenses-and-copyright.

We require you to either (1) present written permission from the copyright holder to publish these figures specifically under the CC BY 4.0 license, or (2) remove the figures from your submission:

1. You may seek permission from the original copyright holder of Figures S6.1 and S6.2 to publish the content specifically under the CC BY 4.0 license.

We recommend that you contact the original copyright holder with the Content Permission Form (http://journals.plos.org/plosone/s/file?id=7c09/content-permission-form.pdf) and the following text:

“I request permission for the open-access journal PLOS ONE to publish XXX under the Creative Commons Attribution License (CCAL) CC BY 4.0 (http://creativecommons.org/licenses/by/4.0/). Please be aware that this license allows unrestricted use and distribution, even commercially, by third parties. Please reply and provide explicit written permission to publish XXX under a CC BY license and complete the attached form.”

Please upload the completed Content Permission Form or other proof of granted permissions as an "Other" file with your submission.

In the figure caption of the copyrighted figure, please include the following text: “Reprinted from [ref] under a CC BY license, with permission from [name of publisher], original copyright [original copyright year].”

2. If you are unable to obtain permission from the original copyright holder to publish these figures under the CC BY 4.0 license or if the copyright holder’s requirements are incompatible with the CC BY 4.0 license, please either i) remove the figure or ii) supply a replacement figure that complies with the CC BY 4.0 license. Please check copyright information on all replacement figures and update the figure caption with source information. If applicable, please specify in the figure caption text when a figure is similar but not identical to the original image and is therefore for illustrative purposes only.

If the reviewer comments include a recommendation to cite specific previously published works, please review and evaluate these publications to determine whether they are relevant and should be cited. There is no requirement to cite these works unless the editor has indicated otherwise.

Response: Thank you for highlighting the copyright requirements for the Supporting Information figures. Figures S6.1 and S6.2 have been removed from the Supporting Information to comply with PLOS ONE copyright and licensing policies. We have also revised the Methods section to clarify that the promotional and educational materials were reviewed and refined during the stakeholder consultation but are not included as Supporting Information.

Comment 6: The manuscript repeatedly states the consultation was “not qualitative research” but simultaneously uses discussions, field notes, structured worksheets, descriptive synthesis, and “member checking.” This creates conceptual confusion.

Response: We acknowledge that several approaches used in the stakeholder consultation, including facilitated discussions, field notes, descriptive synthesis, and stakeholder validation, share similarities with qualitative engagement approaches and may have created conceptual ambiguity in the original manuscript. To improve clarity, we have revised the manuscript to more clearly describe the activity as an implementation-informed stakeholder engagement activity conducted to support pre-implementation planning and refinement of the pharmacy-led PrEP service delivery model.

While the consultation incorporated discussion-based stakeholder engagement approaches, it was not designed or conducted as a formal qualitative research study intended to generate theory-driven or generalizable qualitative findings. We also revised the manuscript to clarify the purpose and synthesis of stakeholder inputs and replaced the term “member checking” with “feedback, accuracy checking, and validation” to better reflect the stakeholder review process used in this consultation. Relevant revisions were made throughout the Materials and methods, including ethical approval section, to improve conceptual clarity and methodological transparency.

Comment 7: Limited analytical depth. The “Results” section is largely descriptive listing of barriers and solutions. There is: no prioritization framework, no ranking of implementation barriers, no systematic coding structure, no explicit implementation determinant mapping (e.g., CFIR domains), limited synthesis beyond narrative summaries. For implementation science audiences, this weakens rigor. The discussion repeatedly states phrases like: “This reflects an implementation determinant…” This becomes formulaic and reads somewhat AI-assisted/editorially repetitive. The manuscript could likely be reduced by 20–25% without losing substance.

Response: We acknowledge that the stakeholder consultation was designed as a pragmatic, implementation-informed consultation to support pre-implementation planning and refinement of the pharmacy-led PrEP service delivery model, rather than as a formal determinant-mapping or theory-generating implementation science study. Accordingly, the Results section focused on summarizing stakeholder-proposed solutions and synthesizing key implementation considerations relevant to real-world service planning.

To address concerns regarding analytical depth, we clarified in the Methods, Results, and Discussion that the consultation built on findings from a preceding exploratory phase that identified barriers and facilitators to pharmacy-led PrEP implementation, whereas the consultation itself was intended to inform implementation planning and refine the service model for pilot implementation. We also removed terminology such as “implementation determinant” that could imply formal determinant identification or mapping and revised the Discussion to focus on stakeholder-informed implementation considerations rather than implementation framework interpretation.

To strengthen synthesis, we clarified how the key implementation considerations were derived from recurring issues identified across multiple consultation activities, including discussions on proposed solutions, workflow design, pilot procedures, assessment tools, training requirements, and site preparation. We also revised Table 1 (title and legend) to better reflect the relationship between barriers identified during the preceding exploratory phase and stakeholder-proposed solutions, and to explicitly note that the consultation did not include formal prioritization or ranking of barriers or solutions. We also expanded the limitations section to explicitly acknowledge the absence of formal prioritization methods and qualitative coding procedures, and to discuss their implications for interpretation of the findings.

Finally, we improved clarity and readability throughout the Results and Discussion by reducing repetitive implementation science phrasing, streamlining narrative summaries, strengthening integration of stakeholder inputs across implementation considerations, and condensing several descriptive sections. These revisions were undertaken to better align the methodological approach, analysis, and interpretation with the pragmatic and formative purpose of the consultation.

Comment 8: Potential selection bias. Stakeholders were heavily drawn from implementation partners already committed to the pilot: Alpro Pharmacy, DOC2US,

collaborating clinicians/NGOs. This risks: confirmation bias, overly favorable feasibility perceptions, underrepresentation of dissenting regulatory or conservative pharmacy perspectives. The limitation section acknowledges this, but only briefly.

I would want deeper critical reflection. The manusc

Attachment Submitted filename: 1. Response to Reviewers 25062026.docx

# Peer review: Decision Letter 1

**DOI:** 10.1371/journal.pone.0357920.r003

**Authors:** Ali Haider Mohammed

27 Jul 2026

Dear Dr. Altice,

Please submit your revised manuscript by Sep 10 2026 11:59PM. If you will need more time than this to complete your revisions, please reply to this message or contact the journal office at plosone@plos.org. When you're ready to submit your revision, log on to https://www.editorialmanager.com/pone/ and select the 'Submissions Needing Revision' folder to locate your manuscript file.

- A letter that responds to each point raised by the academic editor and reviewer(s). You should upload this letter as a separate file labeled 'Response to Reviewers'.
- A marked-up copy of your manuscript that highlights changes made to the original version. You should upload this as a separate file labeled 'Revised Manuscript with Track Changes'.
- An unmarked version of your revised paper without tracked changes. You should upload this as a separate file labeled 'Manuscript'.

If you would like to make changes to your financial disclosure, please include your updated statement in your cover letter. Guidelines for resubmitting your figure files are available below the reviewer comments at the end of this letter.

If applicable, we recommend that you deposit your laboratory protocols in protocols.io to enhance the reproducibility of your results. Protocols.io assigns your protocol its own identifier (DOI) so that it can be cited independently in the future. For instructions see: https://journals.plos.org/plosone/s/submission-guidelines#loc-laboratory-protocols. Additionally, PLOS ONE offers an option for publishing peer-reviewed Lab Protocol articles, which describe protocols hosted on protocols.io. Read more information on sharing protocols at https://plos.org/protocols?utm_medium=editorial-email&utm_source=authorletters&utm_campaign=protocols.

As the corresponding author, your ORCID iD is verified in the submission system and will appear in the published article. PLOS supports the use of ORCID, and we encourage all coauthors to register for an ORCID iD and use it as well. Please encourage your coauthors to verify their ORCID iD within the submission system before final acceptance, as unverified ORCID iDs will not appear in the published article. *Only*  the individual author can complete the verification step; PLOS staff *cannot*  verify ORCID iDs on behalf of authors.

We look forward to receiving your revised manuscript.

Kind regards,

Ali Haider Mohammed

Academic Editor

PLOS One

Journal Requirements:

1. If the reviewer comments include a recommendation to cite specific previously published works, please review and evaluate these publications to determine whether they are relevant and should be cited. There is no requirement to cite these works unless the editor has indicated otherwise.

2. Please review your reference list to ensure that it is complete and correct. If you have cited papers that have been retracted, please include the rationale for doing so in the manuscript text, or remove these references and replace them with relevant current references. Any changes to the reference list should be mentioned in the rebuttal letter that accompanies your revised manuscript. If you need to cite a retracted article, indicate the article’s retracted status in the References list and also include a citation and full reference for the retraction notice.

**Additional Editor Comments:**

Please consider adding a brief table summarizing stakeholder characteristics (stakeholder group, number of participants, and role). This would improve transparency and allow readers to better appreciate the diversity of perspectives included in the consultation.

Table 1 is comprehensive but difficult to navigate. Consider indicating which short-term recommendations were ultimately incorporated into the pilot implementation (e.g., using shading, symbols, or an additional column), making it easier for readers to distinguish implemented versus proposed recommendations.

The description of the pharmacist training programme could be strengthened by briefly stating the anticipated delivery format (e.g., online modules, workshops, simulation exercises) or expected duration, even if detailed information is provided in the supplementary materials.

The Discussion could be strengthened by briefly commenting on the applicability of the proposed implementation model to other middle-income countries in Southeast Asia, as this would enhance the broader relevance of the findings beyond Malaysia.

Please consider acknowledging that implementation priorities identified during the consultation may evolve over time as national HIV policies, PrEP delivery models, and regulatory frameworks continue to develop.

The text within Figures 1–3 appears relatively small and may be difficult to read in the final published PDF. Increasing the font size would improve figure readability.

[Note: HTML markup is below. Please do not edit.]

Reviewers' comments:

Reviewer's Responses to Questions

**Comments to the Author**

Reviewer #1: All comments have been addressed

Reviewer #2: All comments have been addressed

**********

2. Is the manuscript technically sound, and do the data support the conclusions?

Reviewer #1: Yes

Reviewer #2: Yes

**********

3. Has the statistical analysis been performed appropriately and rigorously?

Reviewer #1: Yes

Reviewer #2: Yes

**********

4. Have the authors made all data underlying the findings in their manuscript fully available?

The PLOS Data policy

Reviewer #1: Yes

Reviewer #2: Yes

**********

5. Is the manuscript presented in an intelligible fashion and written in standard English?

Reviewer #1: Yes

Reviewer #2: Yes

**********

Reviewer #1: Thank you to the authors for their thorough revision. The manuscript has improved substantially, and my major concerns have been addressed.

The revised Methods and limitations now clearly explain the involvement of community-based organisations and the limited participation of end users. The clarification that Ministry of Health specialists attended as prescribers, rather than in a programmatic or regulatory role, also resolves the earlier inconsistency.

The revised feasibility language is now appropriately cautious, describing the model as perceived by stakeholders to be feasible. It is also helpful that the Results clarify that no formal prioritisation or scoring was used.

The new discussion of affordability is particularly valuable. The estimated monthly cost of RM157 is higher than the amount many Malaysian men who have sex with men are willing to pay, with more than half preferring a cost below RM100. The authors may wish to mention this gap briefly in the abstract, as it could affect the model’s sustainability.

I have two minor suggestions:

The Methods state that a summary report was circulated to stakeholders for validation. Please consider reporting how many stakeholders responded and whether their feedback led to any substantive changes.

Although a formal qualitative reporting framework was not used, the authors could consider providing the five-item GRIPP2-SF checklist as supporting information if the editor finds it useful.

Reviewer #2: 1. Clarify why GRIPP2-SF/SRQR/COREQ were not applicable.

>Suggestion: Given that the consultation was conducted as an implementation-informed stakeholder engagement activity rather than a qualitative research study involving formal qualitative data collection and analysis, we considered SRQR and COREQ not directly applicable; similarly, GRIPP2-SF was not adopted because the activity was not designed as a patient and public involvement initiative.

2. Ensure every use of "feasible," "agreed," "prioritized," and "acceptable" is appropriately qualified.

3. Clarify exactly what the RM157/month estimated cost includes. Does this cover:

PrEP medication?

consultation?

HIV testing?

renal function testing?

pharmacy service fee?

telemedicine fee?

4. Define more explicitly how the 10 key implementation considerations were derived or consider explicitly stating that the ten considerations are researcher-derived synthesis categories rather than formally validated qualitative themes.

**********

what does this mean?). If published, this will include your full peer review and any attached files.

If you choose “no”, your identity will remain anonymous but your review may still be made public.

**Do you want your identity to be public for this peer review?**  For information about this choice, including consent withdrawal, please see our Privacy Policy

Reviewer #1: **Yes:** Fahmi Hassan

Reviewer #2: No

**********

[NOTE: If reviewer comments were submitted as an attachment file, they will be attached to this email and accessible via the submission site. Please log into your account, locate the manuscript record, and check for the action link "View Attachments". If this link does not appear, there are no attachment files.]

To ensure your figures meet our technical requirements, please review our figure guidelines: https://journals.plos.org/plosone/s/figures

You may also use PLOS’s free figure tool, NAAS, to help you prepare publication quality figures: https://journals.plos.org/plosone/s/figures#loc-tools-for-figure-preparation.

NAAS will assess whether your figures meet our technical requirements by comparing each figure against our figure specifications.

# Author comment: Author response to Decision Letter 2

**DOI:** 10.1371/journal.pone.0357920.r004

6 Aug 2026

Professor Frederick L. Altice

Professor of Medicine, Epidemiology, and Public Health

Yale School of Medicine

Yale University, United States of America

frederick.altice@yale.edu

5th August 2026

Academic Editor

PLOS ONE

Subject: Response to Reviewers for Manuscript Revision (PONE-D-26-19386R1)

Dear Dr. Ali Haider Mohammed,

We sincerely appreciate the opportunity to revise our manuscript, titled "Stakeholder-informed pre-implementation refinement of a pharmacy-led pre-exposure prophylaxis (PrEP) service delivery model in Malaysia" (Manuscript ID: PONE-D-26-19386R1).

We are grateful for the constructive feedback provided by the academic editor and reviewers. We have carefully addressed each comment and have made the necessary revisions to enhance the clarity and quality of our manuscript. Below, we provide a point-by-point response to each comment, detailing the changes made.

________________________________________

Journal requirements:

Comment 1: If the reviewer comments include a recommendation to cite specific previously published works, please review and evaluate these publications to determine whether they are relevant and should be cited. There is no requirement to cite these works unless the editor has indicated otherwise.

Response: We have reviewed and evaluated all reviewer-recommended publications for their relevance to our study. Relevant publications have been incorporated into the revised manuscript where appropriate.

Comment 2: Please review your reference list to ensure that it is complete and correct. If you have cited papers that have been retracted, please include the rationale for doing so in the manuscript text, or remove these references and replace them with relevant current references. Any changes to the reference list should be mentioned in the rebuttal letter that accompanies your revised manuscript. If you need to cite a retracted article, indicate the article’s retracted status in the References list and also include a citation and full reference for the retraction notice.

Response: We have reviewed the reference list to ensure that all references are complete, accurate, and formatted according to the journal requirements. We also verified that none of the cited publications are retracted. Minor corrections have been made to the reference list where appropriate.

________________________________________

Additional editor comments:

Comment 1: Please consider adding a brief table summarizing stakeholder characteristics (stakeholder group, number of participants, and role). This would improve transparency and allow readers to better appreciate the diversity of perspectives included in the consultation.

Response: Thank you for this helpful suggestion. We agree that a summary table improves the transparency of the stakeholder consultation. We have added Table 1, which summarizes the stakeholder groups, number of stakeholders, and their roles in the consultation. The table also clarifies overlapping stakeholder roles where applicable. To avoid duplication, we have streamlined the corresponding description in the Methods section and moved the detailed stakeholder information to the table.

Comment 2: Table 1 is comprehensive but difficult to navigate. Consider indicating which short-term recommendations were ultimately incorporated into the pilot implementation (e.g., using shading, symbols, or an additional column), making it easier for readers to distinguish implemented versus proposed recommendations.

Response: We thank the editor for this helpful suggestion. We agree that distinguishing proposed solutions incorporated into the pilot implementation improves the transparency and readability of the table. Rather than adding a column, we have used a check mark (√) to identify proposed solutions that were incorporated into the pilot pharmacy-led PrEP service model and added an explanatory footnote. We have also clarified in the Methods how stakeholders and the research team determined which proposed short-term solutions would be incorporated into the pilot implementation. Finally, we streamlined the wording of several proposed solutions to improve readability and conciseness while preserving the intent of the stakeholder-generated solutions.

Comment 3: The description of the pharmacist training programme could be strengthened by briefly stating the anticipated delivery format (e.g., online modules, workshops, simulation exercises) or expected duration, even if detailed information is provided in the supplementary materials.

Response: We appreciate this suggestion. To provide additional context while maintaining the focus of the manuscript on the stakeholder consultation, we have expanded the Discussion to briefly describe the pharmacist PrEP training programme that was subsequently developed based on the stakeholder recommendations. Specifically, we now state that the programme comprised self-paced online learning followed by an in-person workshop to support pilot implementation, and we refer readers to our previously published paper describing the development and evaluation of the online training component [Ref. 30].

Comment 4: The Discussion could be strengthened by briefly commenting on the applicability of the proposed implementation model to other middle-income countries in Southeast Asia, as this would enhance the broader relevance of the findings beyond Malaysia.

Response: Thank you for this valuable suggestion. We agree that discussing the broader applicability of the proposed implementation model enhances the relevance of our findings beyond the Malaysian context. We have therefore expanded the Discussion to comment on the potential applicability of the model to other middle-income countries in Southeast Asia, while emphasising that transferability will depend on country-specific regulatory frameworks, healthcare financing, pharmacy infrastructure, and the availability of laboratory and referral services. We also clarify that the Malaysian model should be viewed as a contextually adaptable implementation approach rather than a directly transferable service model.

Comment 5: Please consider acknowledging that implementation priorities identified during the consultation may evolve over time as national HIV policies, PrEP delivery models, and regulatory frameworks continue to develop.

Response: Thank you for highlighting this important point. We agree that acknowledging the evolving implementation landscape strengthens the broader applicability of our findings. We have therefore expanded the Discussion to acknowledge that the implementation considerations identified through this stakeholder consultation reflect the policy, regulatory, and service delivery context at the time of the consultation. We also note that their relative relevance may evolve as national HIV policies, PrEP delivery models, prescribing arrangements, and regulatory frameworks continue to develop, and that periodic stakeholder reassessment will be important to ensure that the service delivery model remains responsive to changing healthcare needs and implementation contexts.

Comment 6: The text within Figures 1–3 appears relatively small and may be difficult to read in the final published PDF. Increasing the font size would improve figure readability.

Response: Thank you for highlighting this. We have revised Figures 1–3 by increasing the font size and making minor layout adjustments where necessary to improve readability in the final published PDF.

________________________________________

Reviewer’s comments:

Reviewer's Responses to Questions

Comments to the Author

1. If the authors have adequately addressed your comments raised in a previous round of review and you feel that this manuscript is now acceptable for publication, you may indicate that here to bypass the “Comments to the Author” section, enter your conflict of interest statement in the “Confidential to Editor” section, and submit your "Accept" recommendation.

Reviewer #1: All comments have been addressed

Reviewer #2: All comments have been addressed

________________________________________

2. Is the manuscript technically sound, and do the data support the conclusions?

The manuscript must describe a technically sound piece of scientific research with data that supports the conclusions. Experiments must have been conducted rigorously, with appropriate controls, replication, and sample sizes. The conclusions must be drawn appropriately based on the data presented.

Reviewer #1: Yes

Reviewer #2: Yes

________________________________________

3. Has the statistical analysis been performed appropriately and rigorously?

Reviewer #1: Yes

Reviewer #2: Yes

________________________________________

4. Have the authors made all data underlying the findings in their manuscript fully available?

The PLOS Data policy requires authors to make all data underlying the findings described in their manuscript fully available without restriction, with rare exception (please refer to the Data Availability Statement in the manuscript PDF file). The data should be provided as part of the manuscript or its supporting information, or deposited to a public repository. For example, in addition to summary statistics, the data points behind means, medians and variance measures should be available. If there are restrictions on publicly sharing data—e.g. participant privacy or use of data from a third party—those must be specified.

Reviewer #1: Yes

Reviewer #2: Yes

________________________________________

5. Is the manuscript presented in an intelligible fashion and written in standard English?

PLOS ONE does not copyedit accepted manuscripts, so the language in submitted articles must be clear, correct, and unambiguous. Any typographical or grammatical errors should be corrected at revision, so please note any specific errors here.

Reviewer #1: Yes

Reviewer #2: Yes

________________________________________

6. Review Comments to the Author

Response to Reviewer #1:

Reviewer #1: Thank you to the authors for their thorough revision. The manuscript has improved substantially, and my major concerns have been addressed.

The revised Methods and limitations now clearly explain the involvement of community-based organisations and the limited participation of end users. The clarification that Ministry of Health specialists attended as prescribers, rather than in a programmatic or regulatory role, also resolves the earlier inconsistency.

The revised feasibility language is now appropriately cautious, describing the model as perceived by stakeholders to be feasible. It is also helpful that the Results clarify that no formal prioritisation or scoring was used.

Comment 1: The new discussion of affordability is particularly valuable. The estimated monthly cost of RM157 is higher than the amount many Malaysian men who have sex with men are willing to pay, with more than half preferring a cost below RM100. The authors may wish to mention this gap briefly in the abstract, as it could affect the model’s sustainability.

Response: We thank the reviewer for this helpful suggestion. We agree that affordability is important to the sustainability of pharmacy-led PrEP delivery. After consideration, we retained the cost comparison in the Discussion rather than adding it to the Abstract because the willingness-to-pay estimate was derived from a separate Malaysian study using data collected in 2016. We have expanded the Discussion to clarify the components of the estimated service cost and to note that direct comparison should be interpreted cautiously given changes in economic conditions, PrEP awareness and healthcare financing over time.

I have two minor suggestions:

Comment 2: The Methods state that a summary report was circulated to stakeholders for validation. Please consider reporting how many stakeholders responded and whether their feedback led to any substantive changes.

Response: Thank you for this helpful suggestion. We have clarified the outcome of the stakeholder feedback process in the Methods section. A summary report was circulated to all 28 stakeholders for feedback and accuracy checking. One stakeholder acknowledged receipt and did not request changes; no other stakeholders responded. Because of the limited response, this circulation was not interpreted as formal stakeholder validation. The manuscript has been revised accordingly.

Comment 3: Although a formal qualitative reporting framework was not used, the authors could consider providing the five-item GRIPP2-SF checklist as supporting information if the editor finds it useful.

Response: We thank the reviewer for this thoughtful suggestion. We carefully considered providing the GRIPP2-SF checklist. However, we concluded that GRIPP2-SF was not the most appropriate reporting guideline for this study. The stakeholder consultation was conducted to refine the implementation strategy for the pharmacy-led PrEP service by obtaining input from multiple stakeholder groups, including community pharmacists, PrEP prescribers, NGO representatives, telemedicine providers, professional societies, and implementation science researchers. Although NGO representatives participated in the consultation, it was designed as a multi-stakeholder implementation consultation, and participants contributed primarily in their stakeholder roles to inform implementation planning rather than as patients or members of the public acting as partners in the research process. Therefore, we concluded that including the GRIPP2-SF checklist could inadvertently misrepresent the nature of the stakeholder consultation. We have clarified this distinction in the manuscript by describing the stakeholder consultation as a multi-stakeholder consultation conducted to refine the implementation strategy.

________________________________________

Response to Reviewer #2:

Comment 1: Clarify why GRIPP2-SF/SRQR/COREQ were not applicable.

>Suggestion: Given that the consultation was conducted as an implementation-informed stakeholder engagement activity rather than a qualitative research study involving formal qualitative data collection and analysis, we considered SRQR and COREQ not directly applicable; similarly, GRIPP2-SF was not adopted because the activity was not designed as a patient and public involvement initiative.

Response: We agree. Thank you.

Comment 2: Ensure every use of "feasible," "agreed," "prioritized," and "acceptable" is appropriately qualified.

Response: Thank you for this important comment. We have carefully reviewed the manuscript and revised the wording throughout to avoid unqualified use of terms such as "feasible," "agreed," "prioritized," and "acceptable." Where appropriate, we have replaced these terms with more precise language that accurately reflects the stakeholder consultation findings and avoids implying objective conclusions or unanimous consensus.

Comment 3: Clarify exactly what the RM157/month estimated cost includes. Does this cover:

PrEP medication? Consultation? HIV testing? Renal function testing? Pharmacy service fee? Telemedicine fee?

Response: Thank you for highlighting this. We have clarified in the Discussion that the estimated monthly cost of approximately RM157 included the cost of PrEP medication (RM97) and an HIV self-test kit (RM60) provided through the pharmacy during the study period. We also clarified that medication rebates were introduced during the pilot to reduce financial barriers, allowing participants to access PrEP medication at RM65 per month while HIV self-testing was provided at no cost. For clarity, the reported estimate did not include renal function testing, as this was performed according to clinical need rather than at every visit. Consultation fees were not charged in Malaysian community pharmacies, and telemedicine costs were absorbed by participating pharmacies through their subscription arrangements with the telemedicine provider.

Comment 4: Define more explicitly how the 10 key implementation considerations were derived or consider explicitly stating that the ten considerations are researcher-derived synthesis categories rather than formally validated qualitative themes.

Response: Thank you for this important comment. We agree that the derivation of the ten implementation considerations should be

Attachment Submitted filename: 1. Response to Reviewers 05082026.docx

# Editor report: Decision Letter 2

**DOI:** 10.1371/journal.pone.0357920.r005

**Authors:** Ali Haider Mohammed

24 Aug 2026

Stakeholder-informed pre-implementation refinement of a pharmacy-led pre-exposure prophylaxis (PrEP) service delivery model in Malaysia

PONE-D-26-19386R2

Dear Dr. Frederick,

We’re pleased to inform you that your manuscript has been judged scientifically suitable for publication and will be formally accepted for publication once it meets all outstanding technical requirements.

Within one week, you’ll receive an e-mail detailing the required amendments. When these have been addressed, you’ll receive a formal acceptance letter and your manuscript will be scheduled for publication.

An invoice will be generated when your article is formally accepted. Please note, if your institution has a publishing partnership with PLOS and your article meets the relevant criteria, all or part of your publication costs will be covered. Please make sure your user information is up-to-date by logging into Editorial Manager at Editorial Manager® and clicking the ‘Update My Information' link at the top of the page. For questions related to billing, please contact billing support.

If your institution or institutions have a press office, please notify them about your upcoming paper to help maximize its impact. If they’ll be preparing press materials, please inform our press team as soon as possible -- no later than 48 hours after receiving the formal acceptance. Your manuscript will remain under strict press embargo until 2 pm Eastern Time on the date of publication. For more information, please contact onepress@plos.org.

Kind regards,

Ali Haider Mohammed

Academic Editor

PLOS One

Additional Editor Comments (optional):

All comments are well addressed.

Reviewers' comments:

# Editor report: Acceptance letter

**DOI:** 10.1371/journal.pone.0357920.r006

**Authors:** Ali Haider Mohammed

PONE-D-26-19386R2

PLOS One

Dear Dr. Altice,

I'm pleased to inform you that your manuscript has been deemed suitable for publication in PLOS One. Congratulations! Your manuscript is now being handed over to our production team.

At this stage, our production department will prepare your paper for publication. This includes ensuring the following:

* All references, tables, and figures are properly cited

* All relevant supporting information is included in the manuscript submission,

* There are no issues that prevent the paper from being properly typeset

You will receive further instructions from the production team, including instructions on how to review your proof when it is ready. Please keep in mind that we are working through a large volume of accepted articles, so please give us a few days to review your paper and let you know the next and final steps.

Lastly, if your institution or institutions have a press office, please let them know about your upcoming paper now to help maximize its impact. If they'll be preparing press materials, please inform our press team within the next 48 hours. Your manuscript will remain under strict press embargo until 2 pm Eastern Time on the date of publication. For more information, please contact onepress@plos.org.

You will receive an invoice from PLOS for your publication fee after your manuscript has reached the completed accept phase. If you receive an email requesting payment before acceptance or for any other service, this may be a phishing scheme. Learn how to identify phishing emails and protect your accounts at https://explore.plos.org/phishing.

If we can help with anything else, please email us at customercare@plos.org.

Thank you for submitting your work to PLOS One and supporting open access.

Kind regards,

PLOS One Editorial Office Staff

on behalf of

Dr. Ali Haider Mohammed

Academic Editor

PLOS One
